# High two-month mortality in Gabonese adults with and without tuberculosis: A prospective cohort study of patients with presumed pulmonary tuberculosis

**DOI:** 10.1016/j.ijregi.2026.100870

**Published:** 2026-03-01

**Authors:** Stefan Fabian Weber, Anne Glaser, Bayodé Roméo Adegbite, Jabar Babatunde Pacome Agbo Achimi Abdul, Guy Arnault Rogue Mfoumbi Ibinda, Micheska Epola Dibamba Ndanga, Christiane Sidonie Gouleu, Jean-Ulrich Muandze Nzambe, Frank Tobian, Kindie Fentahun Muchie, Mary Gaeddert, Fabrice Mbaïdiguim, Karen Chetcuti, Michael Lanzer, Claudia M. Denkinger, Martin Peter Grobusch, Ayola Akim Adegnika, Sabine Bélard, for the NoTB study group, Andréa Rosine Oméra Obele Ndong, Jean Ronald EDOA, Paul A Nguema Moure, Jenny Mouloungui-Mavoungou, Colombe Backanot, Volker Rickerts, Ilka McCormick, Dennis Nurjadi, Sebastien Boutin, Heike Jung, Cindy Büchler, María del Mar Castro, Emilija Sorgho-Mitreska, Rima Jeske, Tim Waterboer, Elisabeth Mamani-Mategula

**Affiliations:** 10 Centre de Recherches Médicales de Lambaréné (CERMEL); 11 Robert Koch Institute, Berlin; 12 Institute of Medical Microbiology, University Hospital Schleswig-Holstein Campus Lübeck, Germany and German Center for Infection Research (DZIF), Partner Site Hamburg-Lübeck-Borstel-Riems; 13 Heidelberg University Hospital, Department of Parasitology; 14 Heidelberg University Hospital, Department of Infectious Disease and Tropical Medicine; 15 German Cancer Research Center (DKFZ), Division of Infections and Cancer Epidemiology; 16 School of Global and Public Health, Kamuzu University of Health Sciences, Blantyre, Malawi and Training and Research Unit of Excellence (TRUE), Blantyre, Malawi; 1 Heidelberg University Hospital, Department of Infectious Disease and Tropical Medicine, Heidelberg, Germany; 2 Heidelberg University Hospital, Department of Parasitology, Heidelberg, Germany; 3 German Center for Infectious Disease Research (DZIF) partner site Heidelberg, Heidelberg, Germany; 4 Tübingen University Hospital, Institute of Tropical Medicine, Tübingen, Germany; 5 Centre de Recherches Médicales de Lambaréné (CERMEL), Lambaréné, Gabon; 6 Center of Tropical Medicine and Travel Medicine, Amsterdam University Medical Centers, Location University of Amsterdam, Amsterdam, The Netherlands; 7 German Center for Infectious Disease Research (DZIF) partner site Tübingen, Tübingen, Germany; 8 Radiology Division, Department of Paediatrics and Child Health, Kamuzu University of Health Sciences, Blantyre, Malawi; 9 Department of Epidemiology and Biostatistics, School of Public Health, College of Medicine and Health Sciences, Bahir Dar University, Bahir Dar, Ethiopia

**Keywords:** Tuberculosis, Diagnosis, HIV, Mortality, Respiratory infections, Sub-Saharan Africa

## Abstract

**Objectives:**

Pulmonary tuberculosis (PTB) remains a leading cause of mortality in sub-Saharan Africa but often remains unconfirmed. Patient outcomes in unconfirmed TB or non-TB cases are rarely studied. We assessed 2-month outcomes and associated factors in presumed TB cases independent of TB status.

**Methods:**

Hospitalized adults with presumed PTB in Lambaréné, Gabon, were prospectively enrolled (September 2024-January 2025). Demographic data, including a socioeconomic status (SES) score, were collected. Laboratory analyses included HIV-test, GeneXpert and mycobacterial culture. Chest X-rays (CXR) were assessed for signs of TB and non-TB conditions. Diagnoses were categorized as *confirmed PTB, clinically diagnosed PTB*, or *non-TB*. Two-month follow-up assessed outcomes.

**Results:**

Of 103 participants, 30 of 102 (29%) were HIV positive. Median age was 44 years (interquartile range 28-57). PTB was confirmed in 34 of 103 (33%), clinically diagnosed in 5 of 103 (5%), and not diagnosed in 62 of 103 (62%). In the non-TB group, CXR findings were consistent with bacterial pneumonia (24/62, 39%) or malignancy (12/62, 19%). At follow-up, 81 of 100 (81%) reported improvement, whereas 19 of 100 (19%) did not, including 12 of 100 (12%) deaths. HIV infection, smoking, alcohol use, rural residence, and lower SES were associated with death, no difference was seen by TB status.

**Conclusion:**

High 2-month mortality, regardless of final TB status, highlights the need for improved access to diagnostics and better treatment algorithms in severe respiratory illness.

**Study registry:**

German Clinical Trials Register (DRKS00034074).

## Highlights

• Two-month mortality exceeded 10% in hospitalized adults with presumed tuberculosis.
• Mortality was independent from whether tuberculosis was present or not.
• HIV infection, low socioeconomic status, and rural residence were linked to death.
• Most non-TB cases showed bacterial or malignant patterns on chest X-ray.
• Algorithms after negative TB tests need to be improved for better patient outcomes.

## Introduction

Tuberculosis (TB) remains a leading cause of infectious disease-related morbidity and mortality in sub-Saharan Africa, with an estimated 2.5 million new cases and 291,000 deaths annually in the World Health Organization (WHO) African region [1]. In Gabon, TB continues to pose a major public health challenge, with an estimated incidence of 505 cases per 100,000 population, rendering it among the top-10 high TB-burden countries globally [1]. Beyond TB, other conditions can also cause severe lung disease resembling TB [2]. However, knowledge and capacity for diagnosing non-TB etiologies in Africa are often limited (e.g., for fungal diseases [3]), frequently resulting in empiric antibiotic therapy [4].

Pulmonary TB (PTB) diagnosis relies on molecular or culture-based sputum tests (bacteriological confirmation). Diagnostic tools such as the GeneXpert platform have been rolled out on a large scale, but even where available, the diagnostic yield (i.e., the proportion of confirmed diagnosis per person tested [5]) may be limited by factors such as sputum scarcity or paucibacillary disease (e.g., in HIV). Test cartridge stock-outs or infrastructural limitations additionally restrict availability in resource-limited settings [6].

In practice, TB is often diagnosed clinically, based on signs and symptoms and supported by radiological findings. In Gabon, only 66% of new or relapse PTB cases were bacteriologically confirmed in 2023 [1]. A 2024 systematic review and meta-analysis investigated the frequency of and factors associated with the absence of bacteriological confirmation in empirically treated TB (no test performed or negative, ambiguous, or unclear result) in 85,623 patients. It showed that clinical TB diagnosis accounts for 40% globally, particularly in settings with limited diagnostic capacity, and is associated with increased risk of adverse outcomes, including death [7]. For Gabon, the national estimate for the proportion of unconfirmed TB was 34% in 2023 [1].

### Rationale

TB-centered studies often focus on the outcome of individuals with TB (e.g., in a study assessing treatment outcomes only in TB cases [8], and in Gabon previous research focused on outpatient cohorts [9]. Less attention is given to populations once a *non-TB* diagnosis is assumed, and data on outcomes are lacking.

### Aims

This proof-of-concept study investigated all participants in a cohort of hospitalized adults in Lambaréné, Gabon, with presumed PTB, including those without a subsequent confirmed or clinical PTB diagnosis. While the broader study goal was to characterize non-TB causes of severe respiratory disease, in this first cohort analysis we assessed TB status (PTB confirmed, PTB clinically diagnosed, or non-TB) and evaluated clinical outcomes after 2 months (improvement, deterioration, or death) based on standard-of-care diagnostics complemented by study mycobacterial culture.

## Methods

### Study design and procedures

This was a prospective cohort study conducted at two hospitals in Lambaréné, Gabon (Hôpital Albert Schweitzer; Centre Hospitalier Régional Georges Rawiri de Lambaréné). Hospitalized adults in the internal medicine departments were consecutively screened by chart review or by consultation with healthcare providers. Inclusion criteria were: (i) adults (≥18 years) with clinically and/or radiologically presumed PTB with TB workup initiated by attending physicians (typically one GeneXpert Ultra on sputum and chest X-ray [CXR]); and (ii) additional positive WHO symptom screening (any of the following: cough lasting ≥2 weeks or any duration in people living with HIV [PLHIV], fever, weight loss, night sweats [10]). Patients were excluded if they had already taken anti-TB treatment (ATT) for ≥7 days.

Study procedures included a questionnaire regarding current symptoms, previous medical history, and TB risk factors (HIV, contacts, previous TB). A socioeconomic status (SES) score was calculated by adding points of a 14-item household asset index. Routine laboratory and radiology test results relevant to the presenting symptoms were extracted from medical records. The following additional specimens were collected and tested at the Centre de Recherches Médicales de Lambaréné (CERMEL [11]) laboratories: blood for HIV serology (or polymerase chain reaction if inconclusive), blood count, HbA1c (cut-off 47.5 mmol/mol), and C-reactive protein (CRP, cut-off 5 mg/l); an additional spot sputum for TB liquid culture (BD Bactec MGIT). Study results, including positive TB cultures, were disclosed to attending physicians, acknowledging the potential incorporation bias in patient management. Clinician-indicated CXR hard-copy films were photographed and reviewed using a structured case report form by a radiologist with >13 years of experience in TB and HIV care in sub-Saharan Africa, blinded to clinical data and study tests. Abnormalities in the lung parenchyma (by zone and type), airways, mediastinum, cardiac silhouette, pleura, and soft tissues/bones were recorded. CXRs were classified as typical for TB with subcategorization as either primary, post-primary, or post-TB sequelae, untypical or uncertain for TB (primary, post-primary, or post-TB sequelae); additionally, abnormal CXRs were assigned alternative (non-TB) diagnoses if consistent pathology was present.

Additional testing for non-TB pathogens on other samples (cf. study registry DRKS00034074) is beyond the scope of this analysis and will be reported separately.

Participants were contacted after 2 months for a telephonic follow-up inquiring about the evolution of presenting signs and symptoms. Participants were categorized based on self-report as *improvement* (total recovery, significant recovery, at least some recovery), *no improvement or worsening* (symptoms unchanged, deterioration, or death), and *lost to follow-up* (unreachable).

### TB reference standard

TB status was determined as *confirmed PTB* (GeneXpert Ultra or culture positive, regardless of whether ATT was initiated or not), *clinical PTB* (start of ATT without criteria for *confirmed PTB)*, or *non-TB* (negative TB test and no ATT during hospitalization as per patient file or as per telephone follow-up).

### Outcomes

The outcomes for this analysis were (i) patient outcomes at 2-month follow-up; (ii) the TB status: the proportion of confirmed or clinical PTB and those not diagnosed with TB; and (iii) subgroup analyses across groups of patient outcome, HIV, and TB status. Further subgroup analyses for the rural/urban status and the SES are provided in Supplement Table 1a-e.

### Statistical analyses

The sample size was calculated for the primary outcome of non-TB pathogen prevalence, which does not apply to this analysis focusing on patient outcomes. The analysis population consisted of all participants recruited; missing data and loss of follow-up are indicated by available cases in respective denominators. The crude household index was calculated and classified into high, medium, and low SES on a quantile basis. Descriptive statistics are provided as medians and interquartile ranges (IQRs) or proportions. Differences between groups were explored for key variables using Firth’s bias-reduced logistic regression to account for a small sample size and potential separation. For binary group comparisons, the method was applied directly. For outcomes with more than two categories, groups were compared through pairwise contrasts, each analyzed with Firth’s regression. Results are reported in Supplement Table 1a-e as two-sided *P*-values, applying a significance level at *P* < 0.05. Upset plots were created for frequent combinations of CXR findings. Analyses and figures were done in R Studio (version 4.4.3; packages: openxlsx, ggplot2, dplyr, UpSetR, logistf, janitor) and Microsoft Excel.

### Ethical aspects and registration

The study was approved by the CERMEL institutional ethics committee (CEI-006/2024, approval 5 August 2024) and conducted in accordance with the Declaration of Helsinki. All participants provided written informed consent. The study is registered in the German Clinical Trials Register (DRKS00034074). This article conforms to the STROBE guidelines for the reporting of observational studies [12], see Supplemental Materials.

## Results

**Figure 1:**
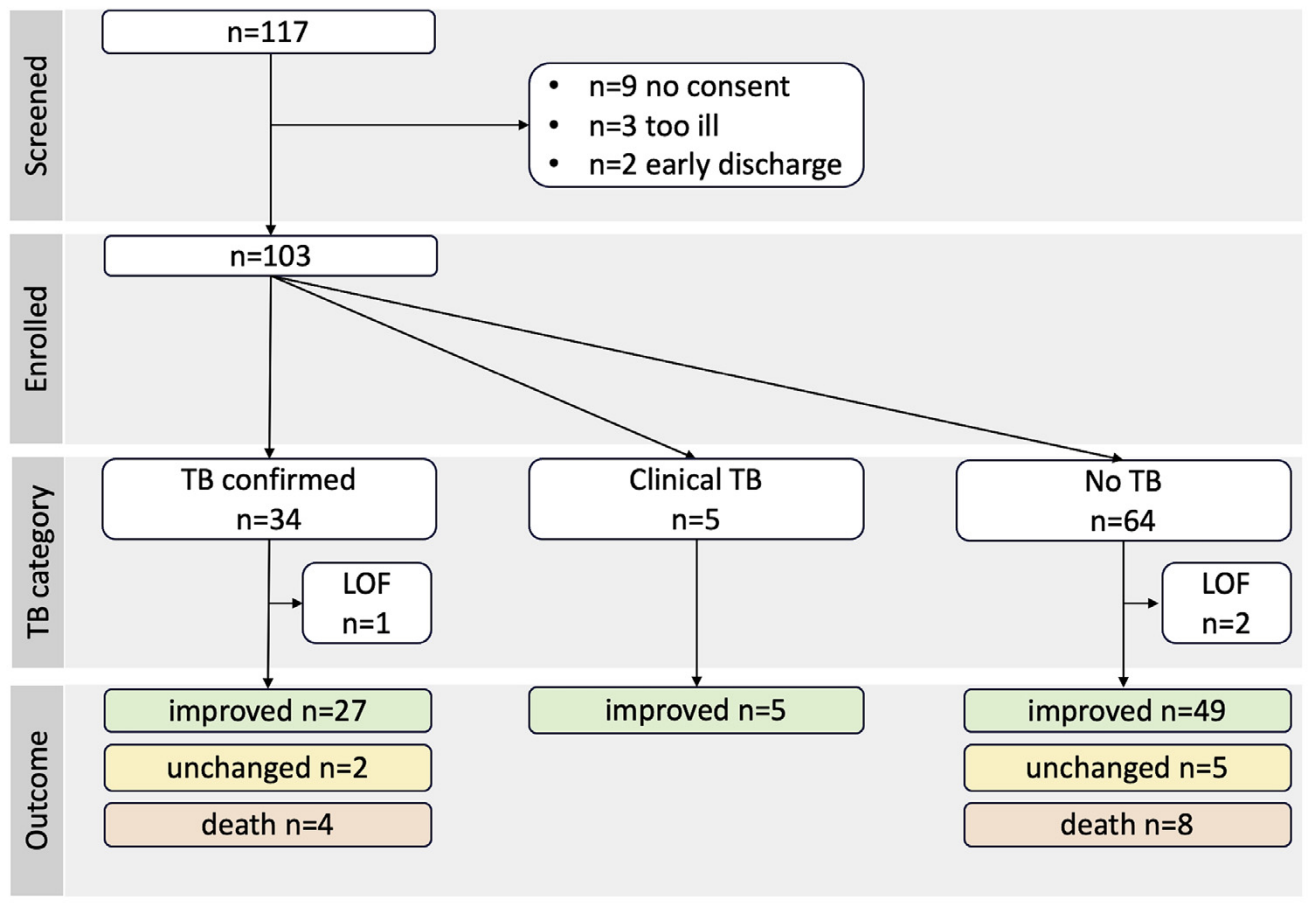
Patient flow chart. PTB confirmed: of 29 GeneXpert-positive cases, 25 (86%) received ATT, three participants died before ATT initiation, and one was referred for multi-drug-resistant treatment; 1of 5 (20%) culture-positive case died before ATT initiation, 1 of 5 (20%) had already received empirical ATT, and 3 of 5 (60%) had not started ATT at time of culture-positivity; clinical PTB: all cases received ATT. ATT, anti-TB treatment; LOF, loss of follow-up (n = 3 not reached); PTB, pulmonary TB; TB, tuberculosis.

Recruitment took place between 19 September 2024 and 31 January 2025, and follow-up was completed in April 2025. Overall, 117 patients were screened, of whom 103 were enrolled (Figure 1).

### Cohort description

Participant data is provided in Table 1a, Table 1b. Of 103 enrolled participants, 61 of 103 (59%) were female. The median age was 44 years, and 30 of 102 (29%) were PLHIV; most (19 of 30, 63%) were diagnosed during the current hospitalization and 8 of 30 (27%) were on anti-retroviral therapy (ART) before hospitalization (4 of 8 for ≤3 months). A third of participants smoked tobacco (33 of 103, 32%; 25 of 33, 76% were male), with a median 6.25 pack-years and 40 of 101 (40%) reported consuming alcohol at least weekly. Diabetes was uncommon with 4 of 82 (5%, where HbA1c available). A previous TB episode was reported by 19 of 102 (19%), but only 13 of 19 (68%) of these reported ATT durations of at least 6 months. The most common symptoms were cough (97 of 103, 94%) and weight loss (87 of 103, 84%). Laboratory analyses showed leukocytosis >10/nL in 25 of 93 (27%) and elevated CRP >5 mg/l in 96 of 101 (95%) participants. Participants originated mostly from urban areas (72/103, 70%) around Lambaréné. Eighty participants provided information for the SES assessment. Scores ranged from 0-10 (median 4, IQR 3-6); 31 of 80 (39%) were stratified into the low SES group, 24 of 80 (30%) into medium, and 25 of 80 (31%) into high.

**Table 1a:** Cohort characteristics stratified by patient outcome.

All participants (n = 103)			Patient outcome^c^ n = 100		
			*Improvement* *n = 81*	*No improvement or worsening (including deceased)* *n = 19*	*Deceased^b^* *n = 12*
Demographics and risk factors					
**Age (years) median**[**IQR]**		44 [30.5-58.5] (N = 103)	42 [28-57] (N = 81)	52 [35-67.5] (N = 19)	48.5 [38.5-63.75] (N = 12)
**Sex female, n (%)**		61/103 (59)	51/81 (63)	9/19 (47)	6/12 (50)
**Weight (kg) median**[**IQR]**		55.5 [45-64] (N = 100)^a^	55.5 [46.5-64] (N = 80)	56 [44-64] (N = 17)	49 [41-58.5] (N = 11)
**HIV test result positive, n (%)**		30/102 (29)	25/81 (31)	5/19 (26)	5/11 (45)
**Previously known PLHIV, currently on ART, n (%)**		8/11 (73)	6/8 (75)	2/3 (67)	2/3 (67)
**Smoking, n (%)**		33/103 (32)	**21/81 (26)**	**10/19 (53)**	**8/12 (67)**
**Alcohol use (≥1x/week), n (%)**		40/101 (40)	30/81 (37)	9/17 (53)	7/10 (70)
**Previous TB episode, n (%)**		19/102 (19)	14/80 (18)	5/19 (26)	3/12 (25)
**Previous ATT (≥6 months), n (%)**		13/19 (68)	10/14 (71)	3/5 (60)	2/3 (67)
**Known TB contact, n (%)**		23/103 (22)	21/81 (26)	2/19 (11)	1/12 (8)
**Known TB contact current, n (%)**		6/103 (6)	6/81 (7)	0/19 (0)	0/12 (0)
Social determinants					
**Urban residence, n (%)**		72/103 (70)	**60/81 (74)**	**11/19 (58)**	**5/12 (42)**
**Social score^e^^,^ median (IQR)**		4 [3-6] (N = 80) ^f^	**5 [3-6] (N = 61)**	**4 [2-5.25] (N = 16)**	**3 [1.25-4] (N = 10)**
**Social low, n (%)^e^**		31/80 (39)	22/61 (36)	7/16 (44)	6/10 (60)
**Social medium, n (%)^e^**		24/80 (30)	19/61 (31)	5/16 (31)	3/10 (30)
**Social high, n (%)^e^**		25/80 (31)	20/61 (33)	4/16 (25)	1/10 (10)
Symptoms					
**Cough, n (%)**		97/103 (94)	77/81 (95)	17/19 (89)	11/12 (92)
**Duration: median weeks**[**IQR]**		3 [1-4] (N = 97)	3 [1-4] (N = 77)	3 [2-8] (N = 17)	4 [2-7] (N = 11)
**Fever, n (%)**		81/103 (79)	**68/81 (84)**	**11/19 (58)**	**8/12 (67)**
**Duration: median weeks**[**IQR]**		1 [0.75-3] (N = 81)	1 [0.5-3] (N = 68)	2 [1.5-5] (N = 11)	2 [1.75-4.5] (N = 8)
**Weight loss n (%)**		87/103 (84)	68/81 (84)	16/19 (84)	11/12 (92)
**Duration: median weeks**[**IQR]**		4 [2-12] (N = 82)	4 [2-8] (N = 63)	7 [2-12] (N = 16)	8 [3-32] (N = 11)
**Night sweats, n (%)**		33/103 (32)	29/81 (36)	4/19 (21)	3/12 (25)
**Duration: median weeks**[**IQR]**		2 [1-4] (N = 33)	2 [1-4] (N = 29)	2.5 [1.75-4.25] (N = 4)	2 [1.5-5] (N = 3)
**Hemoptysis, n (%)**		31/103 (30)	25/81 (31)	5/19 (26)	5/12 (42)
**Duration: median weeks**[**IQR]**		0.5 [0.4-2] (N = 31)	0.5 [0.3-2] (N = 25)	1 [1-4] (N = 5)	1 [1-4] (N = 5)
**Dyspnea, n (%)**		62/103 (60)	44/81 (54)	15/19 (79)	11/12 (92)
**Duration: median weeks**[**IQR]**		2 [1-4] (N = 62)	1.5 [1-4] (N = 44)	3 [1.5-7] (N = 15)	4 [1.5-7] (N = 11)
**Fatigue, n (%)**		71/103 (69)	53/81 (65)	17/19 (89)	12/12 (100)
**Duration: median weeks**[**IQR]**		2 [1-4] (N = 71)	2 [1-4] (N = 53)	4 [1-8] (N = 17)	4 [2.5-6.5] (N = 12)
Laboratory					
**WBC/nL, median**[**IQR]**		7.72 [5.38-10.4] (N = 93)	**7.78 [5.48-9.79] (N = 74)**	**7.19 [4.91-13.14] (N = 16)**	**11.6 [6.64-14.2] (N = 9)**
**WBC ≥10/nL, n (%)**		25/93 (27)	**17/74 (23)**	**7/16 (44)**	**5/9 (56)**
**Neutrophile of WBC %, median [IQR]**		58.93 [45.87-69.42] (N = 91)	59.19 [45.31-67.96] (N = 72)	55.55 [50.94-73.04] (N = 16)	67.36 [51.65-71.23] (N = 9)
**CRP mg/l, median**[**IQR]**		71.31 [41.65-133.11] (N = 101)	75.38 [43.08-133.11] (N = 81)	70.66 [46.86-116.97] (N = 17)	70.98 [65.88-171.03] (N = 10)
**CRP >5 mg/l, n (%)**		96/101 (95)	79/81 (98)	16/17 (94)	9/10 (90)
**HbA1c >47.5 mmol/mol, n (%)**		4/82 (5)	3/66 (5)	1/14 (7)	1/8 (12)
Radiology					
**CXR abnormal, n (%) ^g^**		88/101 (87)	69/79 (87)	17/19 (89)	10/12 (83)
**Number of parenchymal zones affected by pathology, median**[**IQR]**		2 [1-4] (N = 101)	2 [1-3.5] (N = 79)	3 [1.5-4] (N = 19)	3 [3-4.25] (N = 12)
**CXR typical for TB? n (%)**	Yes	31/101 (31)	25/79 (32)	5/19 (26)	3/12 (25)
	Uncertain	3/101 (3)	2/79 (3)	1/19 (5)	1/12 (8)
	No	67/101 (66)	52/79 (66)	13/19 (68)	8/12 (67)
**CXR consistent with non-TB etiology, n (%)^h^**	Bacterial	43/101 (43)	32/79 (41)	11/19 (58)	6/12 (50)
	Viral	11/101 (11)	7/79 (9)	4/19 (21)	3/12 (25)
	Fungal	3/101 (3)	2/79 (3)	1/19 (5)	1/12 (8)
	Cardiac	7/101 (7)	5/79 (6)	2/19 (11)	0/12 (0)
	Malignant	21/101 (21)	17/79 (22)	3/19 (16)	2/12 (17)
	Structural lung pathology	2/101 (2)	1/79 (1)	0/19 (0)	0/12 (0)
	Atelectasis	1/101 (1)	1/79 (1)	0/19 (0)	0/12 (0)
	Unspecific	1/101 (1)	1/79 (1)	0/19 (0)	0/12 (0)
TB status					
**PTB confirmed, n (%)^d^**		34/103 (33)	27/81 (33)	6/19 (32)	4/12 (33)
**Clinical PTB, n (%)**		5/103 (5)	5/81 (6)	0/19 (0)	0/12 (0)
**No TB diagnosed, n (%)**		64/103 (62)	49/81 (60)	13/19 (68)	8/12 (67)

Footnote explanations are provided under Table 1b.

**Table 1b:** Cohort characteristics stratified by TB status.

All participants (n = 103)			TB status n = 103			
			*PTB diagnosed*			*Non-TB* *n = 64*
			*PTB confirmed or clinical* *n = 39*	*PTB confirmed* *n = 34*	*Clinical PTB* *n = 5*	
**Demographics and risk factors**						
**Age (years), median**[**IQR]**		44 [30.5-58.5] (N = 103)	**33 [24-48.5] (N = 39)**	**32.5 [23.25-46.5] (N = 34)**	**58 [42-59] (N = 5)**	**48 [36.75-63.25] (N = 64)**
**Sex female, n (%)**		61/103 (59)	24/39 (62)	21/34 (62)	3/5 (60)	37/64 (58)
**Weight (kg). median**[**IQR]**		55.5 [45-64] (N = 100)^a^	**50 [42-58] (N = 38)**	**50 [42-57] (N = 33)**	**58 [57-64] (N = 5)**	**58.5 [50-65] (N = 62)**
**HIV test result positive, n (%)**		30/102 (29)	12/38 (32)	9/33 (27)	3/5 (60)	18/64 (28)
**Previously known PLHIV, currently on ART, n (%)**		8/11 (73)	4/5 (80)	2/3 (67)	2/2 (100)	4/6 (67)
**Smoking, n (%)**		33/103 (32)	10/39 (26)	8/34 (24)	2/5 (40)	23/64 (36)
**Alcohol consumption (≥1x/week), n (%)**		40/101 (40)	14/39 (36)	11/34 (32)	3/5 (60)	26/62 (42)
**Previous TB episode, n (%)**		19/102 (19)	8/39 (21)	7/34 (21)	1/5 (20)	11/63 (17)
**Previous ATT (≥6 months), n (%)**		13/19 (68)	5/8 (62)	4/7 (57)	1/1 (100)	8/11 (73)
**Known TB contact, n (%)**		23/103 (22)	9/39 (23)	8/34 (24)	1/5 (20)	14/64 (22)
**Known TB contact current, n (%)**		6/103 (6)	2/39 (5)	2/34 (6)	0/5 (0)	4/64 (6)
Social determinants						
**Urban residence, n (%)**		72/103 (70)	30/39 (77)	26/34 (76)	4/5 (80)	42/64 (66)
**Social score**^e^**median [IQR**]		4 [3-6] (N = 80)^f^	5 [3-6.5] (N = 31)	5 [3-6] (N = 26)	6 [5-7] (N = 5)	4 [3-6] (N = 49)
**Social low, n (%)**^e^		31/80 (39)	10/31 (32)	9/26 (35)	1/5 (20)	21/49 (43)
**Social medium, n (%)**^e^		24/80 (30)	9/31 (29)	8/26 (31)	1/5 (20)	15/49 (31)
**Social high, n (%)**^e^		25/80 (31)	12/31 (39)	9/26 (35)	3/5 (60)	13/49 (27)
Symptoms						
**Cough, n (%)**		97/103 (94)	38/39 (97)	33/34 (97)	5/5 (100)	59/64 (92)
**Duration: median weeks**[**IQR]**		3 [1-4] (N = 97)	3 [2-4] (N = 38)	3 [1-4] (N = 33)	2 [2-3] (N = 5)	3 [1-5] (N = 59)
**Fever, n (%)**		81/103 (79)	33/39 (85)	28/34 (82)	5/5 (100)	48/64 (75)
**Duration: median weeks**[**IQR]**		1 [0.75-3] (N = 81)	2 [1-3] (N = 33)	2 [1-3] (N = 28)	2 [1-2] (N = 5)	1 [0.5-2] (N = 48)
**Weight loss, n (%)**		87/103 (84)	**37/39 (95)**	**33/34 (97)**	**4/5 (80)**	**50/64 (78)**
**Duration: median weeks**[**IQR]**		4 [2-12] (N = 82)	4 [3-8] (N = 35)	4 [3-8] (N = 32)	4 [3-15] (N = 3)	4 [2-12] (N = 47)
**Night sweats, n (%)**		33/103 (32)	**17/39 (44)**	**14/34 (41)**	**3/5 (60)**	**16/64 (25)**
**Duration: median weeks**[**IQR]**		2 [1-4] (N = 33)	2 [1-4] (N = 17)	3 [1-4] (N = 14)	2 [1.25-4] (N = 3)	2.25 [1-4] (N = 16)
**Hemoptysis, n (%)**		31/103 (30)	15/39 (38)	15/34 (44)	0/5 (0)	16/64 (25)
**Duration: median weeks**[**IQR]**		0.5 [0.4-2] (N = 31)	0.5 [0.4-1.5] (N = 15)	0.5 [0.4-1.5] (N = 15)	NA [NA-NA] (N = 0)	1 [0.45-2.5] (N = 16)
**Dyspnea, n (%)**		62/103 (60)	26/39 (67)	23/34 (68)	3/5 (60)	36/64 (56)
**Duration: median weeks**[**IQR]**		2 [1-4] (N = 62)	2 [1-4] (N = 26)	2 [1-4] (N = 23)	2 [1.5-27] (N = 3)	2 [1-4.5] (N = 36)
**Fatigue, n (%)**		71/103 (69)	25/39 (64)	20/34 (59)	5/5 (100)	46/64 (72)
**Duration: median weeks**[**IQR]**		2 [1-4] (N = 71)	3 [1-4] (N = 25)	4 [1-8] (N = 20)	2 [1-2] (N = 5)	2 [1-4] (N = 46)
Laboratory						
**WBC/nL median**[**IQR]**		7.72 [5.38-10.4] (N = 93)	7.86 [6.24-10.62] (N = 34)	7.83 [6.12-10.7] (N = 29)	8.16 [7.31-8.25] (N = 5)	7.3 [5.04-10.18] (N = 59)
**WBC ≥10/nL, n (%)**		25/93 (27)	10/34 (29)	9/29 (31)	1/5 (20)	15/59 (25)
N**eutrophile of WBC % median**[**IQR]**		58.93 [45.87-69.42] (N = 91)	61.38 [55.81-68.85] (N = 33)	61.38 [55.81-69.8] (N = 29)	62.93 [59.18-65.93] (N = 4)	54.87 [40.47-69.51] (N = 58)
**CRP mg/l median**[**IQR]**		71.31 [41.65-133.11] (N = 101)	76.84 [56.74-133.19] (N = 38)	73.77 [56.56-133.22] (N = 33)	105.25 [90.02-107.39] (N = 5)	67.36 [19.77-127.17] (N = 63)
**CRP >5mg/l, n (%)**		96/101 (95)	38/38 (100)	33/33 (100)	5/5 (100)	58/63 (92)
**HbA1c >47.5mmol/mol, n (%)**		4/82 (5)	2/31 (6)	2/27 (7)	0/4 (0)	2/51 (4)
Radiology						
**CXR abnormal, n (%)**^g^		88/101 (87)	**34/34 (100)**	**34/34 (100)**	**5/5 (100)**	**49/62 (79)**
**Number of parenchymal zones affected by pathology, median**[**IQR]**		2 [1-4] (N = 101)	**3 [2-4] (N = 39)**	**3 [2-4] (N = 34)**	**3 [2-5] (N = 5)**	**2 [1-3] (N = 62)**
**CXR typical for TB, n (%)**	Yes	31/101 (31)	**22/39 (56)**	**20/34 (59)**^I^	**2/5 (40)**	**9/62 (15)**
	Uncertain	3/101 (3)	**2/39 (5)**	**2/34 (6)**	**0/5 (0)**	**1/62 (2)**
	No	67/101 (66)	**15/39 (38)**	**12/34 (35)**^i^	**3/5 (60)**	**52/62 (84)**
**CXR consistent with non-TB etiology, n (%)**^h^	Bacterial	43/101 (43)	19/39 (49)	16/34 (47)	3/5 (60)	24/62 (39)
	Viral	11/101 (11)	3/39 (8)	3/34 (9)	0/5 (0)	8/62 (13)
	Fungal	3/101 (3)	3/39 (8)	3/34 (9)	0/5 (0)	0/62 (0)
	Cardiac	7/101 (7)	2/39 (5)	2/34 (6)	0/5 (0)	5/62 (8)
	Malignant	21/101 (21)	9/39 (23)	6/34 (18)	3/5 (60)	12/62 (19)
	Structural lung pathology	2/101 (2)	0/39 (0)	0/34 (0)	0/5 (0)	2/62 (3)
	Atelectasis	1/101 (1)	0/39 (0)	0/34 (0)	0/5 (0)	1/62 (2)
	Unspecific	1/101 (1)	0/39 (0)	0/34 (0)	0/5 (0)	1/62 (2)
Patient outcome						
**Improvement, n (%)**		81/100 (81)	32/38 (84)	27/33 (82)	5/5 (100)	49/62 (79)
**Unchanged or worsened (incl. deceased), n (%)**		19/100 (19)	6/38 (16)	6/33 (18)	0	13/62 (21)
**Deceased, n (%)**		12/100 (12)	4/38 (11)	4/33 (12)	0	8/62 (13)

ART, anti-retroviral therapy; ATT, anti-TB treatment; CRP, C-reactive protein; CXR, chest x-ray; IQR, interquartile range; NA, not applicable; PLHIV, people living with HIV; PTB, pulmonary TB; TB, tuberculosis; WBC, white blood cell count.

Significant group comparisons (*P* <0.05) for improvement vs death, PLHIV vs HIV-uninfected and confirmed PTB vs non-TB are marked as bold. All *P*-values are provided in Supplement Table 1.

a Height only available for a minority of participants thus not provided. ^b^Included in *no improvement or worsening* column; in non-TB cases, hospital diagnoses were: pneumonia (n = 4), anemia (n = 2), post-TB sequelae (n = 1), cardiac decompensation (n = 1), liver cirrhosis (n = 1), cerebral toxoplasmosis (n = 1, clinical diagnosis), malaria (n = 1); individual patient data provided in Supplement Table 2. ^c^ Loss of follow-up 3/103 (3%). ^d^ n = 103 participants provided at least one spot sputum for GeneXpert testing, n = 100 provided at least two spot sputa (additional sample for liquid culture); n = 1 case Xpert result was available only after 2-month follow-up due to cartridge shortage, symptoms were unchanged; n = 1 case was discharged before Xpert result was available, unclear if ATT was started, participant deceased at 2-month follow-up.

e Social score variables (cell phone, fridge, freezer, television, car, air condition, cemented house floor, electricity, water meter, flushing toilet, radio, washing machine, water tap in the house, water tap outside the house), each contributing one point to the individual social score, categorization into low (0-3, lowest third of participants), medium (4-5, medium third) and high (6-10, highest third).

f Median (IQR) social score for urban participants: 4 (3;6) (n = 72); for rural participants 4 (2.5;6) (n = 23).

g CXR quality satisfactory in 91/101 (90%), suboptimal in 10/101 (10%), none were non-diagnostic.

h Multiple selection possible.

i Outcome in cases with confirmed PTB depending on whether their CXR was typical or not typical for TB: non-typical CXR: 2/12 (17%) died, 3/12 (25%) unfavorable, 9/12 (75%) favorable; typical CXR: 1/19 (5%) died, 2/19 (11%) unfavorable, 17/19 favorable (89%).

### Hospital treatment and diagnosis

Information on treatment (often initiated before TB tests were available) was available for 74 participants. All but one (73 of 74, 99%) received antibiotic treatment, most commonly amoxicillin (± clavulanic acid; 56 of 74, 76%), or cotrimoxazole (14 of 74, 19%). Fluconazole was the only anti-fungal prescribed in 7 of 74 (9%) participants. A total of 18 of 74 (23%) received antimalarials, and 14 of 74 (19%) received systemic steroids.

The most common discharge diagnoses (available for n = 77 overall and n = 54 non-TB cases) were “pneumopathy,” “lung infiltrate” or “pneumonia” (38 of 54; 70%), malaria (11 of 54, 20%), anemia (7 of 54, 13%), HIV (5 of 54, 9%), and pleuritis (4 of 54, 7%).

### Outcomes

**Figure 2:**
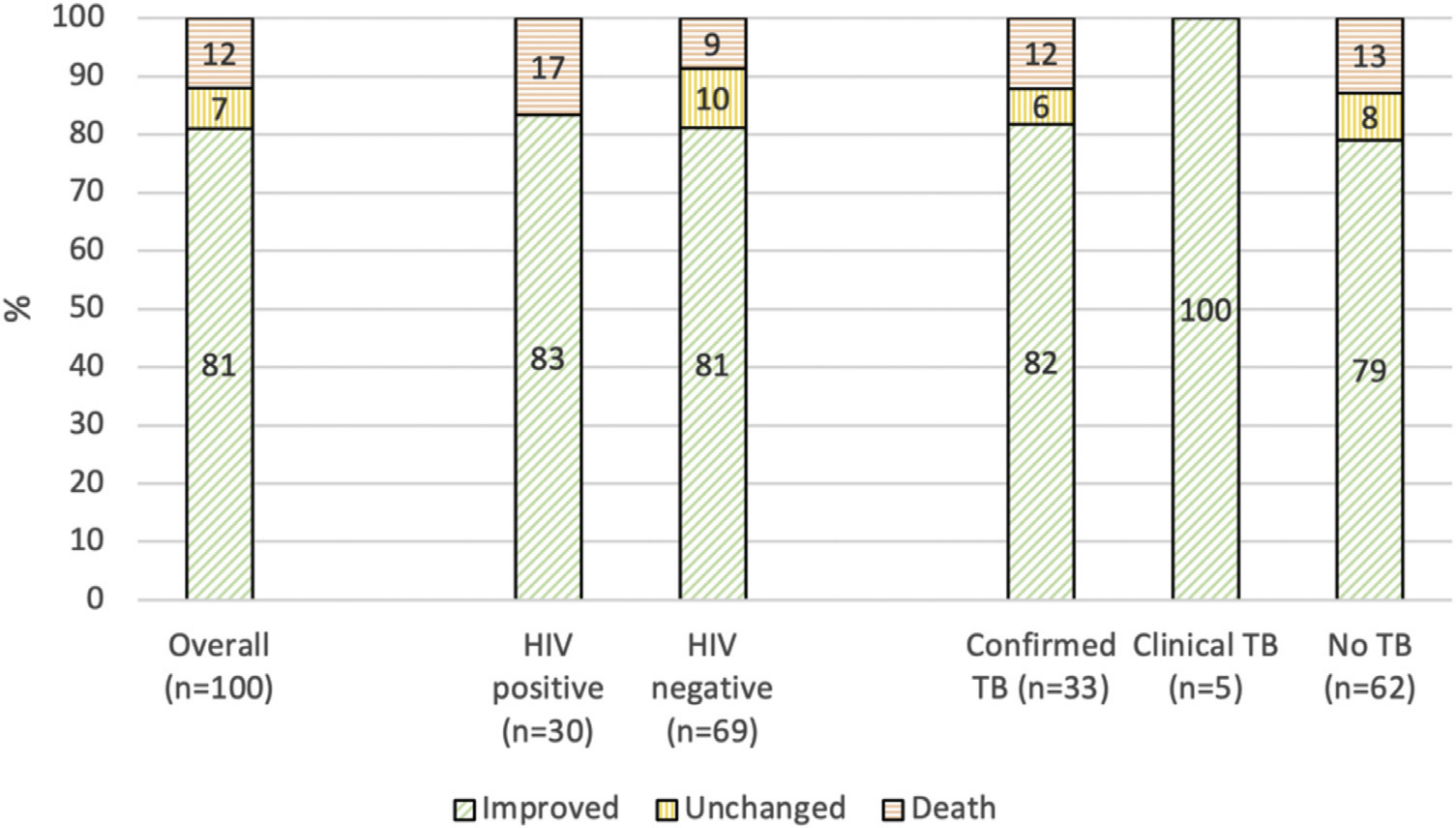
Patient outcomes at 2-month follow-up. Proportion of outcomes in % per category; denominator: participants with available follow-up data (loss to follow-up: n = 3 overall, n = 3 in HIV negative, n = 1 in confirmed PTB, n = 2 in no TB). PTB, pulmonary TB; TB, tuberculosis.

A total of 100 of 103 (97%) participants were reached for follow-up. Recovery was reported as complete (33 of 100, 33%), significant (32 of 100, 32%), or incomplete (16 of 100, 16%). In 7 of 100 participants (7%), symptoms had not changed, and 12 of 100 (12%) had died (Figure 2). Of the deceased participants, 3 of 12 (25%) died during their current admission, and the remainder died after discharge, see Supplement Table 2.

### TB status

A third of the participants (34 of 103, 33%) had confirmed PTB, of whom 29 of 34 (85%) had a positive GeneXpert. Of these, 25 of 29 (86%) received ATT, three participants died before ATT initiation, one was referred for multi-drug-resistant treatment and treatment status remained unknown. In addition, 5 of 34 (15%) were positive by study mycobacterial culture only. These results were communicated to participants and treating physicians; only one participant had received empirical ATT before the test result, and one was started after the result were provided. One participant had already died, and treatment status is unclear for two participants. In summary, 27 of 34 (79%) of confirmed PTB cases received ATT. Rifampicin resistance was detected in 3 of 29 (10%) GeneXpert-positive participants (one with previously successful ATT, one with previously unsuccessful ATT, and one with unclear previous TB status). PTB was diagnosed clinically and treated empirically in 5 of 103 (6%). In 64 of 103 (62%), no TB diagnosis was made.

### CXR interpretation

**Figure 3:**
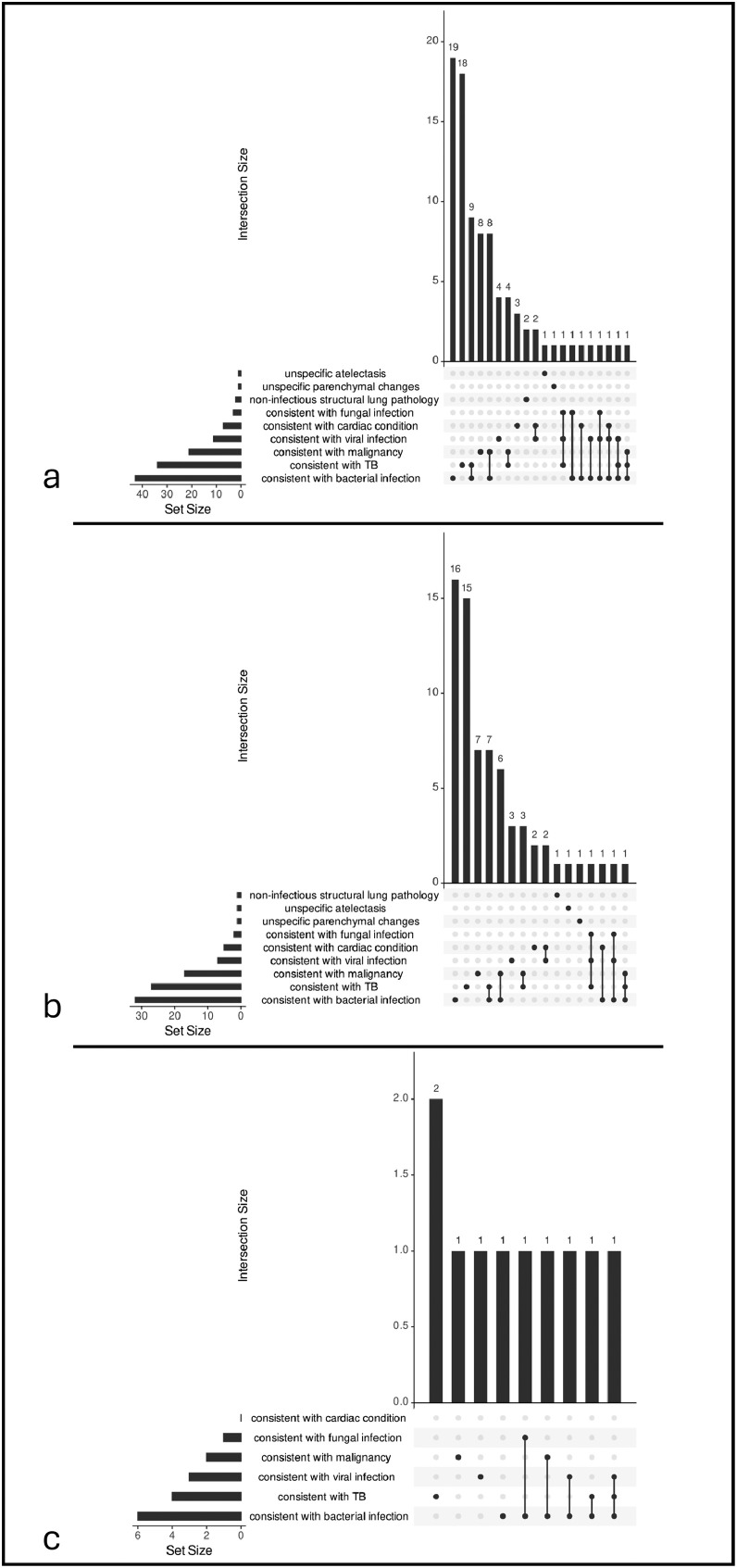
Upset plots for participants with abnormal CXRs. (a) Participants with abnormal lung CXR (n = 87). (b) Participants from a with favorable outcome (n = 68). (c) Participants from a who died (n = 10). CXR, chest X-ray; TB, tuberculosis.

CXR was available in 102 of 103 (99%) participants. Image quality was deemed good in 91 of 101 (90%) and suboptimal in 10 of 101 (10%), but no CXR was judged to be of insufficient quality (Table 1a, b). CXR yielded typical TB findings in 31 of 101 (31%) of cases, whereas in 3 of 101 (3%) TB could not be ruled out, and 67 of 101 (66%) were not typical for TB. Apart from TB, CXR findings were most commonly consistent with bacterial infection (42 of 101, 42%), malignancy (21 of 101, 21%), or viral infection (11 of 101, 11%). CXRs suggestive of bacterial infection were often simultaneously consistent with TB (9 of 42, 21%), or malignancy (8 of 42, 19%), see Figure 3a.

### Comparison of participant characteristics, CXR, and TB status by outcome status

Participants with improvement were younger (42 vs 52 years) and more frequently female (63% vs 47%) than those with no improvement or worsening (Table 1a). Compared with participants with improvement, the following variables were more prevalent or higher in deceased participants: rural vs urban residence (58% vs 26%), HIV prevalence (45% vs 31%), white blood cell counts (11.6/nL vs 7.78/nL), proportions of smoking and alcohol use (smoking: 67% vs 26%; alcohol 70% vs 37%), and proportion of dyspnea (92% vs 54%). Among participants with rural residence, mortality was higher than among those with urban residence (rural: 7 of 29, 24% vs urban: 5 of 71, 7%, see Supplement Table 1d). Participants with lower SES had the highest mortality (high SES: 1 of 24, 4%; medium SES: 3 of 24, 13%; low SES: 6 of 29, 21%), see Supplement Table 1e. For dyspnea and weight loss, symptom duration was twice as long in deceased participants (weight loss: median 8 vs 4 weeks; dyspnea: median 4 vs 1.5 weeks). The following variables were less prevalent or lower in deceased participants compared with participants with improvement: weight (49 kg vs 56 kg), SES score (median 3 [IQR 1.25-4] vs 5 [IQR 3-6]), and fever (58% vs 84%).

Confirmed PTB was equally common in improved and deceased participants (33% each), and no clinical PTB case died.

CXR abnormalities in deceased participants compared with those with a favorable outcome were more frequently consistent with bacterial (6 of 12, 50% vs 31 of79, 39%) and viral infections (3 of 12, 25% vs 7 of 79, 9%). Typical signs for TB were present in 25 of 79 (32%) of participants with favorable outcomes and 5 of 19 (26%) of deceased participants. CXRs consistent with bacterial infection were often also consistent with other conditions: in the favorable outcome group, CXR findings were simultaneously consistent with bacterial infection and TB or malignancy in 7 of 32 (22%) cases each (Figure 3b); in the deceased group, CXR findings were simultaneously consistent with TB or viral infection in 2 of 6 (33%) cases each (Figure 3c). The number of lung zones affected by parenchymal changes was higher in deceased participants than in those with favorable outcome (median 3 [IQR 3-4] vs 2 [IQR 1-3.5]).

### Comparison of participant characteristics, CXR, and outcome by TB status

PTB cases (confirmed or clinical) were younger than the *non-TB* group (33 vs 48 years) and had lower weight (50 kg vs 59 kg), see Table 1b. Smoking was more common in the *non-TB* group, whereas weight loss, night sweats, and hemoptysis were more common in PTB cases. No relevant differences in sex, HIV status, previous TB history, symptom duration, CRP or white blood cell were observed. Also, 2-month mortality did not differ significantly between PTB cases (13%) and *non-TB* (11%). Median SES did not show relevant differences (PTB cases: 5 [IQR 3-6] vs non-TB: 4 [[3], [4], [5], [6]]). Participants with clinical PTB were diagnosed with pleuritis (3 of 5) or pulmonary pathology (2 of 5), and all showed clinical improvement.

Stratifying CXRs by TB status, in confirmed PTB, 20 of 34 (59%) were typical and 12 of 34 (35%) were atypical for TB. In non-TB cases, CXR nevertheless showed typical TB findings in 9 of 62 (15%); CXR findings were consistent with bacterial infection in 24 of 62 (39%), malignancy (12 of 62, 19%), viral infection (8 of 62, 13%) or cardiac conditions (5 of 62, 8%).

### Comparison of participant characteristics, CXR, and TB status by HIV status

PLHIV were more frequently female (73% vs 54%), smoked less commonly (17% vs 38%), and more often reported weight loss (97% vs 79%) and night sweats (50% vs 24%), see Supplement Table 1b. PTB was diagnosed clinically in 3 of 30 (10%) of PLHIV and 2 of 72 (3%) in the HIV-uninfected group. Mortality was higher in PLHIV (17% vs 8%) and occurred during the hospitalization in 1 of 5 (20%) of PLHIV and 2 of 6 (33%) of the HIV-uninfected group (Supplement Table 2). Median SES did not show relevant differences (PLHIV: 4 [IQR 2.25-5] vs HIV-uninfected: 5 [IQR 3-6]). CXR findings were more commonly atypical for TB in PLHIV (PLHIV: 21 of 29, 72% vs HIV-uninfected: 45 of 71, 63%) but were more commonly consistent with bacterial (PLHIV: 17 of 29, 59% vs HIV-uninfected: 24 of 71, 34%) or fungal infection (PLHIV: 2 of 29, 7% vs HIV-uninfected: 1 of 71, 1%).

## Discussion

This study found a high proportion of mortality in patients hospitalized for PTB-suggestive illness in Gabon, which did not differ in patients with confirmed PTB (12%) and those without a TB diagnosis (13%) - a group that is not frequently investigated.

### Mortality

Overall mortality after only 2 months exceeded 10% and was highest in PLHIV (17%), which is in line with previous data from the study site and unchanged since almost 10 years ago [9].

Our exploratory analysis shows that male sex, smoking, HIV infection, rural residence, and lower SES may contribute to poor outcomes or mortality. Possible explanations include late presentation to care due to remote residence and limited access to healthcare, HIV-associated immunosuppression with severe TB disease or alternative or additional opportunistic infections, and predisposing factors for lung pathology such as smoking. Smoking in particular was previously described in Gabonese TB patients at 30% [13] and was slightly more prevalent (38%) in our cohort in *non-TB* cases and 67% in deceased participants. The higher white blood cell and the higher number of lung zones affected by parenchymal changes on CXR in deceased participants may reflect more severe infections. Finally, CXR findings in the deceased group pointed toward bacterial (50%), viral (25%), and fungal (8%) infections as possible non-TB pathogens.

Two-month mortality in confirmed PTB was 12% in our cohort, which had access to GeneXpert testing in the National Reference Centre. However, available evidence remains unclear whether access to GeneXpert testing leads to lower mortality compared with access to smear microscopy only [14], suggesting other risk factors for TB mortality. All deceased cases with confirmed PTB died before start of ATT, and 3 of 4 (75%) within 1 week of hospitalization, suggesting advanced disease. In a systematic review that aimed to describe risks associated with death under anti-TB therapy, the risk for mortality was increased with HIV co-infection, previous TB, and drug-resistant TB [15]. Gabon’s reported TB-associated mortality exceeds 20%, with a combined HIV-positive and -negative mortality rate among incident TB cases of 26% [1]. The slightly lower mortality observed in our cohort likely reflects the short follow-up period and the availability of inpatient care.

Although case numbers were small, in-hospital mortality was 20% in PLHIV and 33% in HIV-uninfected participants (Supplement Table 2), which is within the range reported from inpatient cohorts in high HIV-burden settings [16,17]. Notably, a considerable proportion of deaths occurred after discharge, underscoring the need for strengthened post-discharge follow-up care, particularly for PLHIV. It should also be considered that hospital discharge does not necessarily reflect clinical stabilization and may be influenced by non-medical factors, including patients’ financial constraints. Of participants with a previous history of TB, only 13 of 19 (68%) said to have completed 6 months of ATT, similar to national estimates of treatment success (65% in 2023) [1]. This additionally poses a risk for unfavorable outcomes, recurrent TB, non-TB infections, and the development of drug resistance. Additional patient support during ATT has been shown to improve patient outcomes in a study from South Africa and should also be considered in other settings, such as Gabon [18].

Although participants with negative TB tests would intuitively be assumed to have better outcomes, our data did not show a relevant difference between PTB and *non-TB* mortality (confirmed PTB 12%, *non-TB* 13%), and the proportion of confirmed PTB in *improvement* vs *no improvement or worsening* was similar (33%). Few other studies have investigated patient outcomes after a negative TB test result. A recent TB prevalence study from Zambia and South Africa followed up on participants with abnormal CXR, who were not initiated on ATT. After 9 months, persistent radiological or clinical pathology was present in 43%, and incident TB occurred in only 6%. No data on mortality or underlying pathogenesis were provided. This study also underlines the importance of, and knowledge gaps regarding, unaddressed health conditions in individuals with negative TB tests [19].

### Clinical PTB

Known risk factors for empirical TB treatment (male sex, previous TB, persistent cough, and untreated HIV infection [20]) were prevalent in this study, but notably, clinical PTB status was substantially less prevalent in this cohort (13%) than global estimates from high-burden countries (40% [7]) and national estimates (34% [1]). The reason for this low proportion of clinical PTB may lie in an over-reliance on GeneXpert results in clinical decision-making, where ATT may be withheld after a negative test result. A cautious approach reduces the risk of overtreatment, but underdiagnosis may contribute to missed or delayed treatment in true TB cases.

### Other needs

The proportion of PLHIV with previously unknown status was high (19 of 30, 63%). Even among the 8 of 11 PLHIV with previously known status who were on ART, only one had been treated >1 year, and four had been on ART for ≤3 months. UNAIDS estimated in 2023 that only 58% of PLHIV in Gabon were taking ART, suggesting a need for improved HIV care [21]. Further programmatic data on HIV care are limited, and the proportion of PLHIV with suppressed viral load is not available. In the routine care of our cohort, CD4 cell count testing was not performed for all PLHIV, and information on ART initiation, including timing and proportion among newly diagnosed patients, was not available. One aspect to address mortality and morbidity in hospitalized patients with HIV in this setting may be to include systematic screening for advanced HIV disease using universal CD4 cell count testing, along with early linkage to HIV care and timely ART initiation.

In our cohort, rifampicin resistance was detected in 3 of 29 GeneXpert-positive cases (10%, in line with previous data [22,23]), of whom 2 of 3 had prior incomplete ATT courses (previous TB data unavailable in the third case). These findings point toward a need for improved patient support during ATT to improve retention in care.

Patients often received uniform empiric treatment regimens consisting of broad-spectrum antibiotics, anti-malarial, and anti-fungal agents targeting assumed disease causes, reflecting limitations in local testing capacity for specific pathogens. This practice poses a risk of adverse drug effects in unwarranted treatments and the development of antimicrobial resistance, and high mortality in *non-TB* cases was observed. This highlights a need to improve diagnostic capacity and implement antimicrobial stewardship measures.

Diabetes prevalence was low and in line with previous studies from the region [24]. CRP testing at the proposed cut-off level of 5% for facility-based screening tests was sensitive (100%), but unspecific (8%), suggesting that more adequate screening tools are needed [10].

### Limitations and strengths

The sample size of this proof-of-concept study limits definite conclusions and generalizability. Hypotheses generated here need to be substantiated in larger studies. This analysis includes only TB study testing, allowing insight into the current state of routine care. Future analyses will investigate non-TB infections as possible explanations for poor patient outcomes. During screening, three patients were too sick for recruitment, and four participants were diagnosed with PTB through study tests, which may have biased the outcome to appear better than under non-study conditions. Supply shortages limited testing to one sputum sample per participant; therefore, some PTB cases may have been missed. The recruitment of hospitalized patients may cause bias toward more severe cases but is representative of inpatients with severe respiratory infections. Although the number of clinically diagnosed PTB cases was low, all participants in the *non-TB* group had TB symptoms and might have been empirically treated in other settings, thus allowing some extrapolation to clinical TB status. The SES score has not been validated, but it provides a standardized within-cohort proxy for stratification.

## Conclusion

Our study demonstrates high mortality in both confirmed PTB and patients not diagnosed with PTB despite empirical treatments. Our data identified possible risk factors such as HIV infection, low socioeconomic status, smoking, and alcohol consumption. Exploring clinical pathways to identify care gaps *after* a negative TB test result can inform improved diagnostic and treatment algorithms to support clinicians in identifying probable TB cases requiring empiric ATT or other differential diagnoses based on local epidemiology.

## Acknowledgments

The NoTB study group, in addition to authors listed.

**Table:** 

Centre de Recherches Médicales de Lambaréné (CERMEL)	Andréa Rosine Oméra Obele Ndong Jean Ronald EDOA Paul A Nguema Moure Jenny Mouloungui-Mavoungou Colombe Backanot
Robert Koch Institute, Berlin	Volker Rickerts Ilka McCormick
Institute of Medical Microbiology, University Hospital Schleswig-Holstein Campus Lübeck, Germany and German Center for Infection Research (DZIF), Partner Site Hamburg-Lübeck-Borstel-Riems	Dennis Nurjadi Sebastien Boutin
Heidelberg University Hospital, Department of Parasitology	Heike Jung Cindy Büchler
Heidelberg University Hospital, Department of Infectious Disease and Tropical Medicine	María del Mar Castro Emilija Sorgho-Mitreska
German Cancer Research Center (DKFZ), Division of Infections and Cancer Epidemiology	Rima Jeske Tim Waterboer
School of Global and Public Health, Kamuzu University of Health Sciences, Blantyre, Malawi and Training and Research Unit of Excellence (TRUE), Blantyre, Malawi)	Elisabeth Mamani-Mategula

## Declaration of competing interest

SFW was supported by a DZIF (German Centre for Infectious Disease Research) Clinical Leave stipend (award number 80205CL016) for this project. AG was supported by the Deutsche Studienstiftung with a personal stipend for this project. The authors declare that no conflict of interest exists.

## Appendix Supplementary materials

Image, application 1
