# Supplementary material for: High two-month mortality in Gabonese adults with and without tuberculosis: A prospective cohort study of patients with presumed pulmonary tuberculosis
Source: IJID Reg. 2026 Mar 1;19:100870. doi: 10.1016/j.ijregi.2026.100870 (PMC13022697; doi:10.1016/j.ijregi.2026.100870)
Supplement: Supplementary file 1 [file mmc1.docx]

**SUPPLEMENTAL MATERIAL FOR THE ARTICLE**

**High two-month mortality in Gabonese adults with and without tuberculosis - a prospective cohort study of presumed pulmonary TB patients**

Content

1. Supplement Table 1 – cohort characteristics, extended

a. including p-values for outcome stratification

b. including p-values for HIV stratification

c. including p-values for TB status stratification

d. including stratification for rural/urban residence

e. including stratification for socioeconomic status

2. and rural/urban and socioeconomic status stratification

3. Table 2 – individual patient data of deceased participants

4. STROBE Statement

**Table 1a – cohort characteristics, extended including p-values for outcome stratification**

| **All participants**  **(n=103)** | | | **By patient outcome ^$^**  **n=100** | | | **p-value**  **improved vs. not improved (excluding death)** | **p-value**  **improved vs. death** |
| --- | --- | --- | --- | --- | --- | --- | --- |
|  |  |  | *Improvement*  *n=81* | *No improvement or worsening (incl. deceased)*  *n=19* | *Deceased **  *n=12* |  |  |
| **Demographics and risk factors** | | | | | | | |
| **Age (years) median, IQR** | | 44 [30.5;58.5] (N=103) | 42 [28;57] (N=81) | 52 [35;67.5] (N=19) | 48.5 [38.5;63.75] (N=12) | 0.19 | 0.31 |
| **Sex female n (%)** | | 61/103 (59) | 51/81 (63) | 9/19 (47) | 6/12 (50) | 0.30 | 0.38 |
| **Weight (kg) median, IQR** | | 55.5 (45;64) (N=100) ° | 55.5 [46.5;64] (N=80) | 56 [44;64] (N=17) | 49 [41;58.5] (N=11) | 0.11 | 0.20 |
| **HIV test result positive n (%)** | | 30/102 (29) | 25/81 (31) | 5/19 (26) | 5/11 (45) | 0.20 | 0.32 |
| **Previously known PLHIV currently on ART n (%)** | | 8/11 (73) | 6/8 (75) | 2/3 (67) | 2/3 (67) | ND | |
| **Smoking n (%)** | | 33/103 (32) | 21/81 (26) | 10/19 (53) | 8/12 (67) | 0.76 | 0.01 |
| **Alcohol use (≥ 1x/week) n (%)** | | 40/101 (40) | 30/81 (37) | 9/17 (53) | 7/10 (70) | 0.74 | 0.06 |
| **Previous TB episode n (%)** | | 19/102 (19) | 14/80 (18) | 5/19 (26) | 3/12 (25) | 0.39 | 0.39 |
| **Previous ATT (≥ 6 months) n (%)** | | 13/19 (68) | 10/14 (71) | 3/5 (60) | 2/3 (67) | ND | |
| **Known TB contact n (%)** | | 23/103 (22) | 21/81 (26) | 2/19 (11) | 1/12 (8) | 0.61 | 0.42 |
| **Known TB contact current n (%)** | | 6/103 (6) | 6/81 (7) | 0/19 (0) | 0/12 (0) | ND | |
| **Social determinants** | | | | | | | |
| **Urban residence n (%)** | | 72/103 (70) | 60/81 (74) | 11/19 (58) | 5/12 (42) | 0.65 | 0.03 |
| **Social Score median, IQR "** | | 4 [3;6] (N=80) ^;^ | 5 [3;6] (N=61) | 4 [2;5.25] (N=16) | 3 [1.25;4] (N=10) | 0.04 | 0.07 |
| **Social Low n (%) "** | | 31/80 (39) | 22/61 (36) | 7/16 (44) | 6/10 (60) | ND | |
| **Social Medium n (%) "** | | 24/80 (30) | 19/61 (31) | 5/16 (31) | 3/10 (30) |  |  |
| **Social High n (%) "** | | 25/80 (31) | 20/61 (33) | 4/16 (25) | 1/10 (10) |  |  |
| **Symptoms** | | | | | | | |
| **Cough n (%)** | | 97/103 (94) | 77/81 (95) | 17/19 (89) | 11/12 (92) | 0.18 | 0.42 |
| **Duration: median weeks, IQR** | | 3 [1;4] (N=97) | 3 [1;4] (N=77) | 3 [2;8] (N=17) | 4 [2;7] (N=11) | ND | |
| **Fever n (%)** | | 81/103 (79) | 68/81 (84) | 11/19 (58) | 8/12 (67) | 0.01 | 0.11 |
| **Duration: median weeks, IQR** | | 1 [0.75;3] (N=81) | 1 [0.5;3] (N=68) | 2 [1.5;5] (N=11) | 2 [1.75;4.5] (N=8) | ND | |
| **Weight loss n (%)** | | 87/103 (84) | 68/81 (84) | 16/19 (84) | 11/12 (92) | 0.27 | 0.72 |
| **Duration: median weeks, IQR** | | 4 [2;12] (N=82) | 4 [2;8] (N=63) | 7 [2;12] (N=16) | 8 [3;32] (N=11) | ND | |
| **Night sweats n (%)** | | 33/103 (32) | 29/81 (36) | 4/19 (21) | 3/12 (25) | 0.45 | 0.53 |
| **Duration: median weeks, IQR** | | 2 [1;4] (N=33) | 2 [1;4] (N=29) | 2.5 [1.75;4.25] (N=4) | 2 [1.5;5] (N=3) | ND | |
| **Hemoptysis n (%)** | | 31/103 (30) | 25/81 (31) | 5/19 (26) | 5/12 (42) | 0.20 | 0.43 |
| **Duration: median weeks, IQR** | | 0.5 [0.4;2] (N=31) | 0.5 [0.3;2] (N=25) | 1 [1;4] (N=5) | 1 [1;4] (N=5) | ND | |
| **Dyspnea n (%)** | | 62/103 (60) | 44/81 (54) | 15/19 (79) | 11/12 (92) | 0.91 | 0.04 |
| **Duration: median weeks, IQR** | | 2 [1;4] (N=62) | 1.5 [1;4] (N=44) | 3 [1.5;7] (N=15) | 4 [1.5;7] (N=11) | ND | |
| **Fatigue n (%)** | | 71/103 (69) | 53/81 (65) | 17/19 (89) | 12/12 (100) | 0.88 | 0.08 |
| **Duration: median weeks, IQR** | | 2 [1;4] (N=71) | 2 [1;4] (N=53) | 4 [1;8] (N=17) | 4 [2.5;6.5] (N=12) | ND | |
| **Laboratory** | | | | | | | |
| **WBC /nl median, IQR** | | 7.72 [5.38;10.4] (N=93) | 7.78 [5.48;9.79] (N=74) | 7.19 [4.91;13.14] (N=16) | 11.6 [6.64;14.2] (N=9) | 0.72 | 0.04 |
| **WBC ≥10 /nl n (%)** | | 25/93 (27) | 17/74 (23) | 7/16 (44) | 5/9 (56) | 0.62 | 0.04 |
| **neutrophile of WBC median, IQR** | | 58.93 [45.87;69.42] (N=91) | 59.19 [45.31;67.96] (N=72) | 55.55 [50.94;73.04] (N=16) | 67.36 [51.65;71.23] (N=9) | 0.94 | 0.45 |
| **CRP mg/l median, IQR** | | 71.31 [41.65;133.11] (N=101) | 75.38 [43.08;133.11] (N=81) | 70.66 [46.86;116.97] (N=17) | 70.98 [65.88;171.03] (N=10) | 0.78 | 0.50 |
| **CRP >5mg/l n (%)** | | 96/101 (95) | 79/81 (98) | 16/17 (94) | 9/10 (90) | 0.64 | 0.14 |
| **HbA1c >47.5mmol/mol n (%)** | | 4/82 (5) | 3/66 (5) | 1/14 (7) | 1/8 (12) | 0.83 | 0.22 |
| **Radiology** | | | | | | | |
| **CXR abnormal ^=^** | | 88/101 (87) | 69/79 (87) | 17/19 (89) | 10/12 (83) | 0.54 | 0.65 |
| **Number of parenchymal zones affected by pathology, median, IQR** | | 2 [1;4] (N=101) | 2 [1;3.5] (N=79) | 3 [1.5;4] (N=19) | 3 [3;4.25] (N=12) | 0.65 | 0.11 |
| **CXR typical for TB? n (%)** | Yes  Uncertain  No | 31/101 (31)  3/101 (3)  67/101 (66) | 25/79 (32)  2/79 (3)  52/79 (66) | 5/19 (26)  1/19 (5)  13/19 (68) | 3/12 (25)  1/12 (8)  8/12 (67) | 0.97 | 0.72 ^§^ |
| **CXR consistent with non-TB etiology, n (%) ^+^** | Bacterial  Viral  Fungal  Cardiac  Malignant  Structural lung pathology  Atelectasis  Unspecific | 43/101 (43)  11/101 (11)  3/101 (3)  7/101 (7)  21/101 (21)  2/101 (2)  1/101 (1)  1/101 (1) | 32/79 (41)  7/79 (9)  2/79 (3)  5/79 (6)  17/79 (22)  1/79 (1)  1/79 (1)  1/79 (1) | 11/19 (58)  4/19 (21)  1/19 (5)  2/19 (11)  3/19 (16)  0/19 (0)  0/19 (0)  0/19 (0) | 6/12 (50)  3/12 (25)  1/12 (8)  0/12 (0)  2/12 (17)  0/12 (0)  0/12 (0)  0/12 (0) | ND | |
| **TB status** | | | | | | | |
| **PTB confirmed n (%) ^&^** | | 34/103 (33) | 27/81 (33) | 6/19 (32) | 4/12 (33) | 0.64 | 0.73 ^!^ |
| **Clinical PTB n (%)** | | 5/103 (5) | 5/81 (6) | 0/19 (0) | 0/12 (0) |  |  |
| **No TB diagnosed n (%)** | | 64/103 (62) | 49/81 (60) | 13/19 (68) | 8/12 (67) |  |  |

**Table 1b – cohort characteristics, extended including p-values for HIV stratification**

| **All participants**  **(n=103)** | | | **By HIV status**  **n=102** | | **p-value** |
| --- | --- | --- | --- | --- | --- |
|  |  |  | *PLHIV*  *n=30* | *HIV-uninfected*  *n=72* |  |
| **Demographics and risk factors** | | | | | |
| **Age (years) median, IQR** | | 44 [30.5;58.5] (N=103) | 45 [37.25;55] (N=30) | 41 [25;63.25] (N=72) | 0.92 |
| **Sex female n (%)** | | 61/103 (59) | 22/30 (73) | 39/72 (54) | 0.08 |
| **Weight (kg) median, IQR** | | 55.5 (45;64) (N=100) ° | 50 [41;58] (N=29) | 57 [47.75;64] (N=70) | 0.03 |
| **Smoking n (%)** | | 33/103 (32) | 5/30 (17) | 27/72 (38) | 0.05 |
| **Alcohol use (≥ 1x/week) n (%)** | | 40/101 (40) | 11/29 (38) | 29/72 (40) | 0.85 |
| **Previous TB episode n (%)** | | 19/102 (19) | 7/29 (24) | 12/72 (17) | 0.31 |
| **Previous ATT (≥ 6 months) n (%)** | | 13/19 (68) | 4/7 (57) | 9/12 (75) | ND |
| **Known TB contact n (%)** | | 23/103 (22) | 9/30 (30) | 14/72 (19) | 0.10 |
| **Known TB contact current n (%)** | | 6/103 (6) | 2/30 (7) | 4/72 (6) | ND |
| **Social determinants** | | | | | |
| **Urban residence n (%)** | | 72/103 (70) | 21/30 (70) | 51/72 (71) | 0.90 |
| **Social Score " median, IQR** | | 4 [3;6] (N=80) ^;^ | 4 [2.25;5] (N=26) | 5 [3;6] (N=53) | 0.44 |
| **Social Low n (%) "** | | 31/80 (39) | 11/26 (42) | 19/53 (36) | ND |
| **Social Medium n (%) "** | | 24/80 (30) | 9/26 (35) | 15/53 (28) | ND |
| **Social High n (%) "** | | 25/80 (31) | 6/26 (23) | 19/53 (36) | ND |
| **Symptoms** | | |  | | |
| **Cough n (%)** | | 97/103 (94) | 28/30 (93) | 68/72 (94) | 0.72 |
| **Duration: median weeks, IQR** | | 3 [1;4] (N=97) | 3 [2;4] (N=28) | 3 [1;4] (N=68) | ND |
| **Fever n (%)** | | 81/103 (79) | 25/30 (83) | 55/72 (76) | 0.32 |
| **Duration: median weeks, IQR** | | 1 [0.75;3] (N=81) | 1 [0.5;2] (N=25) | 1 [1;3] (N=55) | ND |
| **Weight loss n (%)** | | 87/103 (84) | 29/30 (97) | 57/72 (79) | 0.07 |
| **Duration: median weeks, IQR** | | 4 [2;12] (N=82) | 5 [3;12] (N=27) | 4 [2;11] (N=54) | ND |
| **Night sweats n (%)** | | 33/103 (32) | 15/30 (50) | 17/72 (24) | 0.01 |
| **Duration: median weeks, IQR** | | 2 [1;4] (N=33) | 2 [1;4.5] (N=15) | 3 [2;4] (N=17) | ND |
| **Hemoptysis n (%)** | | 31/103 (30) | 6/30 (20) | 24/72 (33) | 0.20 |
| **Duration: median weeks, IQR** | | 0.5 [0.4;2] (N=31) | 0.75 [0.5;1.75] (N=6) | 0.5 [0.3;2.25] (N=24) | ND |
| **Dyspnea n (%)** | | 62/103 (60) | 16/30 (53) | 45/72 (62) | 0.39 |
| **Duration: median weeks, IQR** | | 2 [1;4] (N=62) | 2 [0.92;4] (N=16) | 2 [1;4] (N=45) | ND |
| **Fatigue n (%)** | | 71/103 (69) | 23/30 (77) | 47/72 (65) | 0.19 |
| **Duration: median weeks, IQR** | | 2 [1;4] (N=71) | 2 [1;6] (N=23) | 2 [1;4] (N=47) | ND |
| **Laboratory** | | | | | |
| **WBC /nl median, IQR** | | 7.72 [5.38;10.4] (N=93) | 6.97 [4.92;8.61] (N=24) | 7.83 [5.85;11.4] (N=69) | 0.24 |
| **WBC ≥10 /nl n (%)** | | 25/93 (27) | 4/24 (17) | 21/69 (30) | 0.23 |
| **neutrophile of WBC median, IQR** | | 58.93 [45.87;69.42] (N=91) | 60.61 [44.92;69.66] (N=23) | 58.8 [45.92;68.94] (N=68) | 0.64 |
| **CRP mg/l median, IQR** | | 71.31 [41.65;133.11] (N=101) | 73.02 [45.22;119.47] (N=30) | 71.31 [35.11;133.49] (N=71) | 0.92 |
| **CRP >5mg/l n (%)** | | 96/101 (95) | 28/30 (93) | 68/71 (96) | 0.53 |
| **HbA1c >47.5mmol/mol n (%)** | | 4/82 (5) | 1/26 (4) | 3/56 (5) | 0.92 |
| **Radiology** | | | | | |
| **CXR abnormal n (%) ^=^** | | 88/101 (87) | 27/29 (93) | 60/71 (85) | 0.58 |
| **Number of parenchymal zones affected by pathology, median, IQR** | | 2 [1;4] (N=101) | 3 [2;4] (N=29) | 2 [1;3] (N=71) | 0.30 |
| **CXR typical for TB? n (%)** | Yes  Uncertain  No | 31/101 (31)  3/101 (3)  67/101 (66) | 8/29 (28)  0/29 (0)  21/29 (72) | 23/71 (32)  3/71 (4)  45/71 (63) | 0.60 ^§^ |
| **CXR consistent with non-TB etiology, n (%) ^+^** | Bacterial  Viral  Fungal  Cardiac  Malignant  Structural lung pathology  Atelectasis  Unspecific | 43/101 (43)  11/101 (11)  3/101 (3)  7/101 (7)  21/101 (21)  2/101 (2)  1/101 (1)  1/101 (1) | 17/29 (59)  3/29 (10)  2/29 (7)  1/29 (3)  3/29 (10)  0/29 (0)  1/29 (3)  1/29 (3) | 25/71 (35)  7/71 (10)  1/71 (1)  6/71 (8)  18/71 (25)  2/71 (3)  0/71 (0)  0/71 (0) | ND |
| **TB status** | | | | | |
| **PTB confirmed n (%) ^&^** | | 34/103 (33) | 9/30 (30) | 25/72 (35) | 0.80 ^^^ |
| **Clinical PTB n (%)** | | 5/103 (5) | 3/30 (10) | 2/72 (3) |  |
| **No TB diagnosed n (%)** | | 64/103 (62) | 18/30 (60) | 45/72 (62) |  |
| **Patient outcome n (%) ^$^** | | | | | |
| **Improvement n (%)** | | 81/100 (81) | 25/30 (83) | 56/69 (81) | 0.23 ^!^ |
| **Unchanged or worsened (incl. deceased) n (%)** | | 19/100 (19) | 5/30 (17) | 13/69 (19) |  |
| **Deceased n (%)** | | 12/100 (12) | 5/30 (17) | 6/69 (9) |  |

**Table 1c – cohort characteristics, extended including p-values for TB status stratification**

| **All participants**  **(n=103)** | | | **By TB status**  **n=103** | | | | **p-value**  **any TB vs. Non-TB** |
| --- | --- | --- | --- | --- | --- | --- | --- |
|  |  |  | *PTB diagnosed* | | | *Non-TB*  *n=64* |  |
|  |  |  | *PTB confirmed or clinical*  *n=39* | *PTB confirmed*  *n=34* | *Clinical PTB*  *n=5* |  |  |
| **Demographics and risk factors** | | | | | | | |
| **Age (years) median, IQR** | | 44 [30.5;58.5] (N=103) | 33 [24;48.5] (N=39) | 32.5 [23.25;46.5] (N=34) | 58 [42;59] (N=5) | 48 [36.75;63.25] (N=64) | 0.01 |
| **Sex female n (%)** | | 61/103 (59) | 24/39 (62) | 21/34 (62) | 3/5 (60) | 37/64 (58) | 0.72 |
| **Weight (kg) median, IQR** | | 55.5 (45;64) (N=100) ° | 50 [42;58] (N=38) | 50 [42;57] (N=33) | 58 [57;64] (N=5) | 58.5 [50;65] (N=62) | 0.01 |
| **HIV test result positive n (%)** | | 30/102 (29) | 12/38 (32) | 9/33 (27) | 3/5 (60) | 18/64 (28) | 0.80 |
| **Previously known PLHIV currently on ART n (%)** | | 8/11 (73) | 4/5 (80) | 2/3 (67) | 2/2 (100) | 4/6 (67) | ND |
| **Smoking n (%)** | | 33/103 (32) | 10/39 (26) | 8/34 (24) | 2/5 (40) | 23/64 (36) | 0.29 |
| **Alcohol use (≥ 1x/week) n (%)** | | 40/101 (40) | 14/39 (36) | 11/34 (32) | 3/5 (60) | 26/62 (42) | 0.56 |
| **Previous TB episode n (%)** | | 19/102 (19) | 8/39 (21) | 7/34 (21) | 1/5 (20) | 11/63 (17) | 0.72 |
| **Previous ATT (≥ 6 months) n (%)** | | 13/19 (68) | 5/8 (62) | 4/7 (57) | 1/1 (100) | 8/11 (73) | ND |
| **Known TB contact n (%)** | | 23/103 (22) | 9/39 (23) | 8/34 (24) | 1/5 (20) | 14/64 (22) | 0.80 |
| **Known TB contact current n (%)** | | 6/103 (6) | 2/39 (5) | 2/34 (6) | 0/5 (0) | 4/64 (6) | ND |
| **Social determinants** | | | | | | | |
| **Urban residence n (%)** | | 72/103 (70) | 30/39 (77) | 26/34 (76) | 4/5 (80) | 42/64 (66) | 0.24 |
| **Social Score median, IQR "** | | 4 [3;6] (N=80) ^;^ | 5 [3;6.5] (N=31) | 5 [3;6] (N=26) | 6 [5;7] (N=5) | 4 [3;6] (N=49) | 0.26 |
| **Social Low n (%) "** | | 31/80 (39) | 10/31 (32) | 9/26 (35) | 1/5 (20) | 21/49 (43) | ND |
| **Social Medium n (%) "** | | 24/80 (30) | 9/31 (29) | 8/26 (31) | 1/5 (20) | 15/49 (31) | ND |
| **Social High n (%) "** | | 25/80 (31) | 12/31 (39) | 9/26 (35) | 3/5 (60) | 13/49 (27) | ND |
| **Symptoms** | | | | | | | |
| **Cough n (%)** | | 97/103 (94) | 38/39 (97) | 33/34 (97) | 5/5 (100) | 59/64 (92) | 0.36 |
| **Duration: median weeks, IQR** | | 3 [1;4] (N=97) | 3 [2;4] (N=38) | 3 [1;4] (N=33) | 2 [2;3] (N=5) | 3 [1;5] (N=59) | ND |
| **Fever n (%)** | | 81/103 (79) | 33/39 (85) | 28/34 (82) | 5/5 (100) | 48/64 (75) | 0.18 |
| **Duration: median weeks, IQR** | | 1 [0.75;3] (N=81) | 2 [1;3] (N=33) | 2 [1;3] (N=28) | 2 [1;2] (N=5) | 1 [0.5;2] (N=48) | ND |
| **Weight loss n (%)** | | 87/103 (84) | 37/39 (95) | 33/34 (97) | 4/5 (80) | 50/64 (78) | 0.03 |
| **Duration: median weeks, IQR** | | 4 [2;12] (N=82) | 4 [3;8] (N=35) | 4 [3;8] (N=32) | 4 [3;15] (N=3) | 4 [2;12] (N=47) | ND |
| **Night sweats n (%)** | | 33/103 (32) | 17/39 (44) | 14/34 (41) | 3/5 (60) | 16/64 (25) | 0.04 |
| **Duration: median weeks, IQR** | | 2 [1;4] (N=33) | 2 [1;4] (N=17) | 3 [1;4] (N=14) | 2 [1.25;4] (N=3) | 2.25 [1;4] (N=16) | ND |
| **Hemoptysis n (%)** | | 31/103 (30) | 15/39 (38) | 15/34 (44) | 0/5 (0) | 16/64 (25) | 0.15 |
| **Duration: median weeks, IQR** | | 0.5 [0.4;2] (N=31) | 0.5 [0.4;1.5] (N=15) | 0.5 [0.4;1.5] (N=15) | NA [NA;NA] (N=0) | 1 [0.45;2.5] (N=16) | ND |
| **Dyspnea n (%)** | | 62/103 (60) | 26/39 (67) | 23/34 (68) | 3/5 (60) | 36/64 (56) | 0.31 |
| **Duration: median weeks, IQR** | | 2 [1;4] (N=62) | 2 [1;4] (N=26) | 2 [1;4] (N=23) | 2 [1.5;27] (N=3) | 2 [1;4.5] (N=36) | ND |
| **Fatigue n (%)** | | 71/103 (69) | 25/39 (64) | 20/34 (59) | 5/5 (100) | 46/64 (72) | 0.51 |
| **Duration: median weeks, IQR** | | 2 [1;4] (N=71) | 3 [1;4] (N=25) | 4 [1;8] (N=20) | 2 [1;2] (N=5) | 2 [1;4] (N=46) | ND |
| **Laboratory** | | | | | | | |
| **WBC /nl median, IQR** | | 7.72 [5.38;10.4] (N=93) | 7.86 [6.24;10.62] (N=34) | 7.83 [6.12;10.7] (N=29) | 8.16 [7.31;8.25] (N=5) | 7.3 [5.04;10.18] (N=59) | 0.63 |
| **WBC ≥10 /nl n (%)** | | 25/93 (27) | 10/34 (29) | 9/29 (31) | 1/5 (20) | 15/59 (25) | 0.66 |
| **neutrophile of WBC median, IQR** | | 58.93 [45.87;69.42] (N=91) | 61.38 [55.81;68.85] (N=33) | 61.38 [55.81;69.8] (N=29) | 62.93 [59.18;65.93] (N=4) | 54.87 [40.47;69.51] (N=58) | 0.08 |
| **CRP mg/l median, IQR** | | 71.31 [41.65;133.11] (N=101) | 76.84 [56.74;133.19] (N=38) | 73.77 [56.56;133.22] (N=33) | 105.25 [90.02;107.39] (N=5) | 67.36 [19.77;127.17] (N=63) | 0.65 |
| **CRP >5mg/l n (%)** | | 96/101 (95) | 38/38 (100) | 33/33 (100) | 5/5 (100) | 58/63 (92) | 0.18 |
| **HbA1c >47.5mmol/mol n (%)** | | 4/82 (5) | 2/31 (6) | 2/27 (7) | 0/4 (0) | 2/51 (4) | 0.58 |
| **Radiology** | | | | | | | |
| **CXR abnormal n (%) ^=^** | | 88/101 (87) | 34/34 (100) | 34/34 (100) | 5/5 (100) | 49/62 (79) | 0.03 |
| **Number of parenchymal zones affected by pathology, median, IQR** | | 2 [1;4] (N=101) | 3 [2;4] (N=39) | 3 [2;4] (N=34) | 3 [2;5]  (N=5) | 2 [1;3] (N=62) | <0.01 |
| **CXR typical for TB? n (%)** | Yes  Uncertain  No | 31/101 (31)  3/101 (3)  67/101 (66) | 22/39 (56)  2/39 (5)  15/39 (38) | 20/34 (59)  2/34 (6)  12/34 (35) | 2/5 (40)  0/5 (0)  3/5 (60) | 9/62 (15)  1/62 (2)  52/62 (84) | <0.01 ^§^ |
| **CXR consistent with non-TB etiology, n (%) ^+^** | Bacterial  Viral  Fungal  Cardiac  Malignant  Structural lung pathology  Atelectasis  Unspecific | 43/101 (43)  11/101 (11)  3/101 (3)  7/101 (7)  21/101 (21)  2/101 (2)  1/101 (1)  1/101 (1) | 19/39 (49)  3/39 (8)  3/39 (8)  2/39 (5)  9/39 (23)  0/39 (0)  0/39 (0)  0/39 (0) | 16/34 (47)  3/34 (9)  3/34 (9)  2/34 (6)  6/34 (18)  0/34 (0)  0/34 (0)  0/34 (0) | 3/5 (60)  0/5 (0)  0/5 (0)  0/5 (0)  3/5 (60)  0/5 (0)  0/5 (0)  0/5 (0) | 24/62 (39)  8/62 (13)  0/62 (0)  5/62 (8)  12/62 (19)  2/62 (3)  1/62 (2)  1/62 (2) | ND |
| **Patient outcome n (%) ^$^** | | | | | | | |
| **Improvement n (%)** | | 81/100 (81) | 32/38 (84) | 27/33 (82) | 5/5 (100) | 49/62 (79) | 0.77 ^!^ |
| **Unchanged or worsened (incl. deceased) n (%)** | | 19/100 (19) | 6/38 (16) | 6/33 (18) | 0 | 13/62 (21) |  |
| **Deceased n (%)** | | 12/100 (12) | 4/38 (11) | 4/33 (12) | 0 | 8/62 (13) |  |

**Table 1d – cohort characteristics, extended including stratification for rural/urban residence**

| **All participants**  **(n=103)** | | | **By rural or urban residence**  **n=103** | | **p-value** |
| --- | --- | --- | --- | --- | --- |
|  |  |  | *Urban*  *n=72* | *Rural*  *n=31* |  |
| **Demographics and risk factors** | | | | | |
| **Age (years) median, IQR** | | 44 [30.5;58.5] (N=103) | 38.5 [26;56] (N=72) | 54 [39.5;67] (N=31) | 0.01 |
| **Sex female n (%)** | | 61/103 (59) | 43/72 (60) | 18/31 (58) | 0.85 |
| **Weight (kg) median, IQR** | | 55.5 (45;64) (N=100) ° | 57 [47.5;64] (N=71) | 50 [45;63] (N=29) | 0.39 |
| **HIV test result positive n (%)** | | 30/102 (29) | 21/72 (29) | 9/30 (30) | 0.67 |
| **Previously known PLHIV currently on ART n (%)** | | 8/11 (73) | 5/8 (62) | 3/3 (100) | ND |
| **Smoking n (%)** | | 33/103 (32) | 19/72 (26) | 14/31 (45) | 0.06 |
| **Alcohol use (≥ 1x/week) n (%)** | | 40/101 (40) | 24/72 (33) | 16/29 (55) | 0.04 |
| **Previous TB episode n (%)** | | 19/102 (19) | 12/71 (17) | 7/31 (23) | 0.49 |
| **Previous ATT (≥ 6 months) n (%)** | | 13/19 (68) | 6/12 (50) | 7/7 (100) | ND |
| **Known TB contact n (%)** | | 23/103 (22) | 19/72 (26) | 4/31 (13) | 0.20 |
| **Known TB contact current n (%)** | | 6/103 (6) | 4/72 (6) | 2/31 (6) | ND |
| **Social determinants** | | | | | |
| **Social Score median, IQR "** | | 4 [3;6] (N=80) ^;^ | 4 [3;6] (N=57) | 4 [2.5;6] (N=23) | 0.58 |
| **Social Low n (%) "** | | 31/80 (39) | 22/57 (39) | 9/23 (39) | ND |
| **Social Medium n (%) "** | | 24/80 (30) | 18/57 (32) | 6/23 (26) | ND |
| **Social High n (%) "** | | 25/80 (31) | 17/57 (30) | 8/23 (35) | ND |
| **Symptoms** | | | | | |
| **Cough n (%)** | | 97/103 (94) | 68/72 (94) | 29/31 (94) | 0.86 |
| **Duration: median weeks, IQR** | | 3 [1;4] (N=97) | 2.5 [1;4] (N=68) | 4 [2;12] (N=29) | ND |
| **Fever n (%)** | | 81/103 (79) | 59/72 (82) | 22/31 (71) | 0.16 |
| **Duration: median weeks, IQR** | | 1 [0.75;3] (N=81) | 1 [1;2] (N=59) | 1.5 [0.56;4] (N=22) | ND |
| **Weight loss n (%)** | | 87/103 (84) | 60/72 (83) | 27/31 (87) | 0.39 |
| **Duration: median weeks, IQR** | | 4 [2;12] (N=82) | 4 [2;10] (N=57) | 4 [3;12] (N=25) | ND |
| **Night sweats n (%)** | | 33/103 (32) | 24/72 (33) | 9/31 (29) | 0.72 |
| **Duration: median weeks, IQR** | | 2 [1;4] (N=33) | 2.5 [1;4] (N=24) | 2 [2;8] (N=9) | ND |
| **Hemoptysis n (%)** | | 31/103 (30) | 22/72 (31) | 9/31 (29) | 0.88 |
| **Duration: median weeks, IQR** | | 0.5 [0.4;2] (N=31) | 0.5 [0.3;1.75] (N=22) | 1 [0.5;3] (N=9) | ND |
| **Dyspnea n (%)** | | 62/103 (60) | 39/72 (54) | 23/31 (74) | 0.06 |
| **Duration: median weeks, IQR** | | 2 [1;4] (N=62) | 1 [1;4] (N=39) | 4 [1;10] (N=23) | ND |
| **Fatigue n (%)** | | 71/103 (69) | 50/72 (69) | 21/31 (68) | 0.68 |
| **Duration: median weeks, IQR** | | 2 [1;4] (N=71) | 2 [1;4] (N=50) | 4 [2;6] (N=21) | ND |
| **Laboratory** | | | | | |
| **WBC /nl median, IQR** | | 7.72 [5.38;10.4] (N=93) | 7.62 [5.35;9.79] (N=66) | 7.96 [5.97;11.65] (N=27) | 0.45 |
| **WBC ≥10 /nl n (%)** | | 25/93 (27) | 16/66 (24) | 9/27 (33) | 0.37 |
| **neutrophile of WBC median, IQR** | | 58.93 [45.87;69.42] (N=91) | 58.8 [45.68;67.84] (N=64) | 60.94 [45.87;70.48] (N=27) | 0.54 |
| **CRP mg/l median, IQR** | | 71.31 [41.65;133.11] (N=101) | 71.3 [42.36;134.24] (N=71) | 72.54 [36.92;119.72] (N=30) | 0.49 |
| **CRP >5mg/l n (%)** | | 96/101 (95) | 68/71 (96) | 28/30 (93) | 0.61 |
| **HbA1c >47.5mmol/mol n (%)** | | 4/82 (5) | 3/55 (5) | 1/27 (4) | 0.73 |
| **Radiology** | | | | | |
| **CXR abnormal n (%) ^=^** | | 88/101 (87) | 62/71 (87) | 25/30 (83) | 0.37 |
| **Number of parenchymal zones affected by pathology, median, IQR** | | 2 [1;4] (N=101) | 2 [1;3] (N=71) | 3 [2;4.75] (N=30) | 0.08 |
| **CXR typical for TB? n (%)** | Yes  Uncertain  No | 31/101 (31)  3/101 (3)  67/101 (66) | 20/71 (28)  3/71 (4)  48/71 (68) | 11/30 (37)  0/30 (0)  19/30 (63) | 0.40 ^§^ |
| **CXR consistent with non-TB etiology, n (%) ^+^** | Bacterial  Viral  Fungal  Cardiac  Malignant  Structural lung pathology  Atelectasis  Unspecific | 43/101 (43)  11/101 (11)  3/101 (3)  7/101 (7)  21/101 (21)  2/101 (2)  1/101 (1)  1/101 (1) | 30/71 (42)  6/71 (8)  1/71 (1)  5/71 (7)  15/71 (21)  1/71 (1)  1/71 (1)  0/71 (0) | 13/30 (43)  5/30 (17)  2/30 (7)  2/30 (7)  6/30 (20)  1/30 (3)  0/30 (0)  1/30 (3) | ND |
| **TB status** | | | | | |
| **PTB confirmed n (%)  ^&^** | | 34/103 (33) | 26/72 (36) | 8/31 (26) | 0.23 ^^^ |
| **Clinical PTB n (%)** | | 5/103 (5) | 4/72 (6) | 1/31 (3) |  |
| **No TB diagnosed n (%)** | | 64/103 (62) | 42/72 (58) | 22/31 (71) |  |
| **Patient outcome n (%) ^$^** | | | | | |
| **Improvement n (%)** | | 81/100 (81) | 60/71 (85) | 21/29 (72) | 0.02 ^!^ |
| **Unchanged or worsened (incl. deceased) n (%)** | | 19/100 (19) | 11/71 (15) | 8/29 (28) |  |
| **Deceased n (%)** | | 12/100 (12) | 5/71 (7) | 7/29 (24) |  |

**Table 1e– cohort characteristics, extended including stratification for socioeconomic status**

| **All participants**  **(n=103)** | | | **By socioeconomic status**  **n=80** | | | **p-value**  **high vs. medium** | **p-value high vs. low** |
| --- | --- | --- | --- | --- | --- | --- | --- |
|  |  |  | *SES high*  *n=25* | *SES medium*  *n=24* | *SES low*  *n=31* |  |  |
| **Demographics and risk factors** | | | | | | | |
| **Age (years) median, IQR** | | 44 [30.5;58.5] (N=103) | 48 [31;67] (N=25) | 41 [24.75;58.25] (N=24) | 44 [27.5;51] (N=31) | 0.45 | 0.25 |
| **Sex female n (%)** | | 61/103 (59) | 14/25 (56) | 16/24 (67) | 19/31 (61) | 0.45 | 0.69 |
| **Weight (kg) median, IQR** | | 55.5 (45;64) (N=100) ° | 53.5 [48.75;59.92] (N=24) | 54.5 [43.75;65] (N=24) | 50.4 [41.25;63.25] (N=30) | 0.72 | 0.80 |
| **HIV test result positive n (%)** | | 30/102 (29) | 6/25 (24) | 9/24 (38) | 11/30 (37) | 0.32 | 0.33 |
| **Previously known PLHIV currently on ART n (%)** | | 8/11 (73) | 3/4 (75) | 2/3 (67) | 3/4 (75) | ND | |
| **Smoking n (%)** | | 33/103 (32) | 11/25 (44) | 5/24 (21) | 10/31 (32) | 0.10 | 0.37 |
| **Alcohol use (≥ 1x/week) n (%)** | | 40/101 (40) | 10/24 (42) | 12/24 (50) | 10/30 (33) | 0.57 | 0.53 |
| **Previous TB episode n (%)** | | 19/102 (19) | 7/25 (28) | 3/24 (12) | 7/30 (23) | 0.20 | 0.74 |
| **Previous ATT (≥ 6 months) n (%)** | | 13/19 (68) | 6/7 (86) | 1/3 (33) | 5/7 (71) | ND | |
| **Known TB contact n (%)** | | 23/103 (22) | 6/25 (24) | 8/24 (33) | 6/31 (19) | 0.47 | 0.60 |
| **Known TB contact current n (%)** | | 6/103 (6) | 3/25 (12) | 1/24 (4) | 2/31 (6) | ND | |
| **Social determinants** | | | | | | | |
| **Urban residence n (%)** | | 72/103 (70) | 17/25 (68) | 18/24 (75) | 22/31 (71) | 0.60 | 0.81 |
| **Symptoms** | | | | | | | |
| **Cough n (%)** | | 97/103 (94) | 22/25 (88) | 23/24 (96) | 29/31 (94) | 0.38 | 0.49 |
| **Duration: median weeks, IQR** | | 3 [1;4] (N=97) | 4 [2;12] (N=22) | 3 [2;4] (N=23) | 3 [2;4] (N=29) | ND | |
| **Fever n (%)** | | 81/103 (79) | 17/25 (68) | 18/24 (75) | 25/31 (81) | 0.75 | 0.40 |
| **Duration: median weeks, IQR** | | 1 [0.75;3] (N=81) | 3 [2;6] (N=17) | 1 [0.62;2] (N=18) | 1 [1;2] (N=25) | ND | |
| **Weight loss n (%)** | | 87/103 (84) | 22/25 (88) | 21/24 (88) | 27/31 (87) | 0.67 | 0.64 |
| **Duration: median weeks, IQR** | | 4 [2;12] (N=82) | 4.5 [2;12] (N=22) | 4 [2;12] (N=19) | 4 [3;8] (N=26) | ND | |
| **Night sweats n (%)** | | 33/103 (32) | 10/25 (40) | 7/24 (29) | 11/31 (35) | 0.38 | 0.64 |
| **Duration: median weeks, IQR** | | 2 [1;4] (N=33) | 3.5 [2;4.75] (N=10) | 2 [1.5;4] (N=7) | 2.5 [1;4] (N=11) | ND | |
| **Hemoptysis n (%)** | | 31/103 (30) | 7/25 (28) | 7/24 (29) | 8/31 (26) | 0.93 | 0.85 |
| **Duration: median weeks, IQR** | | 0.5 [0.4;2] (N=31) | 1 [0.75;3] (N=7) | 0.5 [0.3;2.5] (N=7) | 0.75 [0.3;1.75] (N=8) | ND | |
| **Dyspnea n (%)** | | 62/103 (60) | 18/25 (72) | 14/24 (58) | 19/31 (61) | 0.33 | 0.42 |
| **Duration: median weeks, IQR** | | 2 [1;4] (N=62) | 4 [2;10] (N=18) | 1.5 [0.62;3.75] (N=14) | 1 [1;4] (N=19) | ND | |
| **Fatigue n (%)** | | 71/103 (69) | 14/25 (56) | 16/24 (67) | 24/31 (77) | 0.56 | 0.14 |
| **Duration: median weeks, IQR** | | 2 [1;4] (N=71) | 3.5 [2;7] (N=14) | 2 [1;4] (N=16) | 3.25 [1;4] (N=24) | ND | |
| **Laboratory** | | | | | | | |
| **WBC /nl median, IQR** | | 7.72 [5.38;10.4] (N=93) | 6.89 [4.27;9.58] (N=24) | 8.7 [6.27;12.52] (N=23) | 7.62 [6.09;9.69] (N=25) | 0.10 | 0.23 |
| **WBC ≥10 /nl n (%)** | | 25/93 (27) | 6/24 (25) | 9/23 (39) | 5/25 (20) | 0.31 | 0.68 |
| **neutrophile of WBC median, IQR** | | 58.93 [45.87;69.42] (N=91) | 55.09 [41.66;67.62] (N=24) | 65.09 [50.45;71.31] (N=23) | 57.34 [44.98;64.24] (N=23) | 0.19 | 0.96 |
| **CRP mg/l median, IQR** | | 71.31 [41.65;133.11] (N=101) | 67.5 [41.65;121.35] (N=25) | 85.61 [56.17;138.96] (N=24) | 78.29 [28.99;133.87] (N=29) | 0.24 | 0.47 |
| **CRP >5mg/l n (%)** | | 96/101 (95) | 24/25 (96) | 23/24 (96) | 26/29 (90) | 0.97 | 0.45 |
| **HbA1c >47.5mmol/mol n (%)** | | 4/82 (5) | 1/19 (5) | 2/23 (9) | 0/23 (0) | 0.74 | 0.42 |
| **Radiology** | | | | | | | |
| **CXR abnormal n (%) ^=^** | | 88/101 (87) | 22/24 (92) | 20/24 (83) | 26/30 (87) | 0.42 | 0.61 |
| **Number of parenchymal zones affected by pathology, median, IQR** | | 2 [1;4] (N=101) | 2 [2;4] (N=24) | 2 [1;3.25] (N=24) | 3 [1.25;3.75] (N=30) | 0.91 | 0.86 |
| **CXR typical for TB? n (%)** | Yes  Uncertain  No | 31/101 (31)  3/101 (3)  67/101 (66) | 9/24 (38)  1/24 (4)  14/24 (58) | 8/24 (33)  0/24 (0)  16/24 (67) | 8/30 (27)  1/30 (3)  21/30 (70) | 0.77 | 0.40 ^§^ |
| **CXR consistent with non-TB etiology, n (%) ^+^** | Bacterial  Viral  Fungal  Cardiac  Malignant  Structural lung pathology  Atelectasis  Unspecific | 43/101 (43)  11/101 (11)  3/101 (3)  7/101 (7)  21/101 (21)  2/101 (2)  1/101 (1)  1/101 (1) | 8/24 (33)  3/24 (12)  0/24 (0)  5/24 (21)  4/24 (17)  0/24 (0)  0/24 (0)  0/24 (0) | 14/24 (58)  3/24 (12)  1/24 (4)  1/24 (4)  6/24 (25)  0/24 (0)  0/24 (0)  1/24 (4) | 14/30 (47)  3/30 (10)  1/30 (3)  0/30 (0)  5/30 (17)  2/30 (7)  1/30 (3)  0/30 (0) | ND | |
| **TB status** | | | | | | | |
| **PTB confirmed n (%)  ^&^** | | 34/103 (33) | 9/25 (36) | 8/24 (33) | 9/31 (29) | 0.47 | 0.24 ^^^ |
| **Clinical PTB n (%)** | | 5/103 (5) | 3/25 (12) | 1/24 (4) | 1/31 (3) |  |  |
| **No TB diagnosed n (%)** | | 64/103 (62) | 13/25 (52) | 15/24 (62) | 21/31 (68) |  |  |
| **Patient outcome n (%) ^$^** | | | | | | | |
| **Improvement n (%)** | | 81/100 (81) | 20/24 (83) | 19/24 (79) | 22/29 (76) | 0.36 | 0.12 ^!^ |
| **Unchanged or worsened (incl. deceased) n (%)** | | 19/100 (19) | 4/24 (17) | 5/24 (21) | 7/29 (24) |  |  |
| **Deceased n (%)** | | 12/100 (12) | 1/24 (4) | 3/24 (13) | 6/29 (21) |  |  |

| **Footnotes for Table 1a-e**  HIV human immunodeficiency virus; IQR interquartile range; PLHIV people living with HIV; ART anti-retroviral therapy: TB tuberculosis; PTB pulmonary tuberculosis; WBC white blood cell count; CRP: C-reactive protein; CXR: chest x-ray; NA not applicable; ND not done  ° height only available for a minority of participants thus not provided  * included in *no improvement or worsening* column*;* in non-TB cases, hospital diagnoses were: pneumonia (n=4), anemia (n=2), post-TB sequelae (n=1), cardiac decompensation (n=1), liver cirrhosis (n=1), cerebral toxoplasmosis (n=1, clinical diagnosis), malaria (n=1); individual patient data provided in Supplement Table 2;  **^$^** loss of follow-up 3/103 (3)  ^&^ n=1 case Xpert result was available only after 2-month follow-up due to cartridge shortage, symptoms were unchanged; n=1 case was discharged before Xpert result was available, unclear if ATT was started, participant deceased at 2-month follow-up  “ social score variables (cell phone, fridge, freezer, television (%) car, air condition, cemented house floor, electricity, water meter, flushing toilet, radio, washing machine, water tap in the house, water tap outside the house), each contributing one point to the individual social score, categorization into low (0-3, lowest third of participants), medium (4-5, medium third) and high (6-10, highest third)  ^;^ median, IQR social score for urban participants: 4 (3;6) (n=72); for rural participants 4 (2.5;6) (n=23)  ^=^ CXR quality satisfactory in 91/101 (90), suboptimal in 10/101 (10), none were non-diagnostic  ^+^ multiple selection possible  outcome in cases with confirmed PTB depending on whether their CXR was typical or not typical for TB: non-typical CXR: 2/12 (17) died, 3/12 (25) unfavorable, 9/12 (75) favorable; typical CXR: 1/19 (5) died, 2/19 (11) unfavorable, 17/19 favorable (89)  ^§^ p-value for CXR typical for TB vs not typical or uncertain  ^!^ p-value for death vs alive  ^^^ p-value for TB confirmed or clinical vs non-TB |
| --- |
|  |

**Supplement Table 2 – individual patient data of deceased participants**

| **Age range** | **Sex** | **TB category** | **Hospital Diagnosis** | **Hospital Treatment** | **Hospital Diagnosis** | **In-hospital death?*** | **HIV status** | **Previous TB** | **Smoking** | **Alcohol use** | **Cough duration (weeks)** | **Hemoptysis** | **Dyspnea** | **fever** | **Night sweats** |
| --- | --- | --- | --- | --- | --- | --- | --- | --- | --- | --- | --- | --- | --- | --- | --- |
| 60s | M | Non-TB | Pneumonia, anemia | Cefaclor, azithromycin, transfusion | Pneumonia, anemia | yes | -ve | no | Yes (20py) | Yes | 4 | No | Yes | No | No |
| 40s | M | Non-TB | Cardiac decompensaton, pneumonia | Amoxi/clav, steroids, furosemide, oxygen | Cardiac decompensaton, pneumonia | no | unknown | No | Yes (unclear) | Unknown | 1 | Yes | Yes | Yes | yes |
| 30s | M | Confirmed TB | TB confirmed, dysglycemia | ATT | TB confirmed, dysglycemia | yes | -ve | No | Yes (3py) | No | 2 | No | Yes | Yes | No |
| 20s | F | Confirmed TB | TB confirmed | ATT, amoxicillin, steroids | TB confirmed | no | -ve | Yes | Yes (2py) | yes | 4 | Yes | Yes | Yes | No |
| 60s | M | Non-TB | Pneumonia, anemia | Amoxi/clav, steroids, transfusion, oxygen | Pneumonia, anemia | no | -ve | No | Yes (10px) | No | 6 | No | Yes | Yes | No |
| 30s | F | Confirmed TB | TB confirmed | ATT not yet started, cotrimoxazole, ciprofloxacin, ART | TB confirmed | no | +ve | No | No | Yes | 3 | No | Yes | No | No |
| 50s | F | Non-TB | Liver cirrhosis | unclear | Liver cirrhosis | no | +ve | No | No | No | 8 | No | Yes | Yes | Yes |
| 30s | M | Non-TB | Cerebral toxoplasmosis (clinical diagnosis) | Cotrimoxazole, fluconoazole | Cerebral toxoplasmosis (clinical diagnosis) | no | +ve | Yes | Yes (<1py) | Yes | 12 | Yes | Yes | Yes | No |
| 80s | F | Non-TB | Pneumonia, malaria (120/µl) | Amoxi/clav, ACT | Pneumonia, malaria (120/µl) | no | -ve | Unknown | Yes (unclear) | Yes | 12 | No | No | No | No |
| 60s | M | Non-TB | Post-TB sequelae | Cefixime, steroids, oxygen | Post-TB sequelae | no | +ve | Yes | Yes (40py) | unclear | 52 | Yes | Yes | No | No |
| 40s | F | Confirmed TB | TB confirmed | Unknown | TB confirmed | yes | +ve | No | No | Yes | 2 | No | Yes | Yes | No |
| 60s | F | Non-TB | unclear | Unknown | unclear | no | -ve | No | No | Yes | 2 | Yes | Yes | Yes | Yes |
| F female, M male; -ve negative; +ve positive; TB tuberculosis; ATT anti-TB treatment; ART antiretroviral therapy  * current hospitalization, possible subsequent hospitalizations in other health-care institutions not ruled out | | | | | | | | | | | | | | | |

**STROBE Statement—checklist of items that should be included in reports of observational studies**

|  | Item No | Recommendation | Location in Text |
| --- | --- | --- | --- |
| **Title and abstract** | 1 | (*a*) Indicate the study’s design with a commonly used term in the title or the abstract | Title |
|  |  | (*b*) Provide in the abstract an informative and balanced summary of what was done and what was found | Abstract |
| Introduction | | | |
| Background/rationale | 2 | Explain the scientific background and rationale for the investigation being reported | Background |
| Objectives | 3 | State specific objectives, including any prespecified hypotheses | Background |
| Methods | | | |
| Study design | 4 | Present key elements of study design early in the paper | Methods: Study design |
| Setting | 5 | Describe the setting, locations, and relevant dates, including periods of recruitment, exposure, follow-up, and data collection | Methods: Study design  Results |
| Participants | 6 | (*a*) *Cohort study*—Give the eligibility criteria, and the sources and methods of selection of participants. Describe methods of follow-up  *~~Case-control study~~*~~—Give the eligibility criteria, and the sources and methods of case ascertainment and control selection. Give the rationale for the choice of cases and controls~~  *~~Cross-sectional study~~*~~—Give the eligibility criteria, and the sources and methods of selection of participants~~ | Methods: Study design |
|  |  | ~~(~~*~~b~~*~~)~~ *~~Cohort study~~*~~—For matched studies, give matching criteria and number of exposed and unexposed~~  *~~Case-control study~~*~~—For matched studies, give matching criteria and the number of controls per case~~ | NA |
| Variables | 7 | Clearly define all outcomes, exposures, predictors, potential confounders, and effect modifiers. Give diagnostic criteria, if applicable | Methods: Study procedures, TB reference standard, Outcomes |
| Data sources/ measurement | 8* | For each variable of interest, give sources of data and details of methods of assessment (measurement). Describe comparability of assessment methods if there is more than one group | Methods: Study procedures |
| Bias | 9 | Describe any efforts to address potential sources of bias | Methods (specifically enrolment procedures, outcome definitions) |
| Study size | 10 | Explain how the study size was arrived at | NA, secondary analysis |
| Quantitative variables | 11 | Explain how quantitative variables were handled in the analyses. If applicable, describe which groupings were chosen and why | Methods: statistical analyses |
| Statistical methods | 12 | (*a*) Describe all statistical methods, including those used to control for confounding | Methods: statistical analyses |
|  |  | (*b*) Describe any methods used to examine subgroups and interactions | Methods: statistical analyses |
|  |  | (*c*) Explain how missing data were addressed | Methods: statistical analyses |
|  |  | (*d*) *Cohort study*—If applicable, explain how loss to follow-up was addressed  *~~Case-control study~~*~~—If applicable, explain how matching of cases and controls was addressed~~  *~~Cross-sectional study~~*~~—If applicable, describe analytical methods taking account of sampling strategy~~ | Methods: statistical analyses |
|  |  | (*e*) Describe any sensitivity analyses | NA |
| Results | | | |
| Participants | 13* | (a) Report numbers of individuals at each stage of study—eg numbers potentially eligible, examined for eligibility, confirmed eligible, included in the study, completing follow-up, and analysed | Results: Figure 1 |
|  |  | (b) Give reasons for non-participation at each stage | Results: Figure 1 |
|  |  | (c) Consider use of a flow diagram | Results: Figure 1 |
| Descriptive data | 14* | (a) Give characteristics of study participants (eg demographic, clinical, social) and information on exposures and potential confounders | Results: Overall cohort |
|  |  | (b) Indicate number of participants with missing data for each variable of interest | Table 1 |
|  |  | (c) *Cohort study*—Summarise follow-up time (eg, average and total amount) | Results: Follow-up outcome |
| Outcome data | 15* | *Cohort study*—Report numbers of outcome events or summary measures over time | Results: Follow-up outcome |
|  |  | *~~Case-control study—~~*~~Report numbers in each exposure category, or summary measures of exposure~~ | NA |
|  |  | *~~Cross-sectional study—~~*~~Report numbers of outcome events or summary measures~~ | NA |
| Main results | 16 | (*a*) Give unadjusted estimates and, if applicable, confounder-adjusted estimates and their precision (eg, 95 confidence interval). Make clear which confounders were adjusted for and why they were included | Subgroup analyses Table 1 |
|  |  | (*b*) Report category boundaries when continuous variables were categorized | Table 1: CRP, HbA1c, WBC |
|  |  | (*c*) If relevant, consider translating estimates of relative risk into absolute risk for a meaningful time period | NA |
| Other analyses | 17 | Report other analyses done—eg analyses of subgroups and interactions, and sensitivity analyses | Results, Table 1 |
| Discussion | | | |
| Key results | 18 | Summarise key results with reference to study objectives | Discussion: mortality |
| Limitations | 19 | Discuss limitations of the study, taking into account sources of potential bias or imprecision. Discuss both direction and magnitude of any potential bias | Discussion: Limitations and strengths |
| Interpretation | 20 | Give a cautious overall interpretation of results considering objectives, limitations, multiplicity of analyses, results from similar studies, and other relevant evidence | Discussion: Conclusion |
| Generalisability | 21 | Discuss the generalisability (external validity) of the study results | Discussion: Limitations and strengths |
| Other information | | | |
| Funding | 22 | Give the source of funding and the role of the funders for the present study and, if applicable, for the original study on which the present article is based | Acknowledgements |
